# Caveolin 1 is a conserved host factor mediating *Bacillus cereus* hemolysin BL toxin cytolytic toxicity

**DOI:** 10.1371/journal.pbio.3003957

**Published:** 2026-08-21

**Authors:** Yingying Lu, Jie Liu, Chengyu Liu, Zehua Zuo, Michael Ewing, Hang Chen, Toren Finkel, Stephen H. Leppla, Shihui Liu

**Affiliations:** 1 Aging Institute of University of Pittsburgh, University of Pittsburgh Medical Center, Pittsburgh, Pennsylvania, United States of America; 2 Division of Cardiology, Department of Medicine, University of Pittsburgh School of Medicine, Pittsburgh, Pennsylvania, United States of America; 3 Transgenic Core Facility, National Heart Lung and Blood Institute, National Institutes of Health, Bethesda, Maryland, United States of America; 4 Microbial Pathogenesis Section, Laboratory of Parasitic Diseases, National Institute of Allergy and Infectious Diseases, National Institutes of Health, Bethesda, Maryland, United States of America; 5 Division of Infectious Diseases, Department of Medicine, University of Pittsburgh School of Medicine, Pittsburgh, Pennsylvania, United States of America; University of California Davis School of Medicine, UNITED STATES OF AMERICA

## Abstract

Hemolysin BL (HBL) is a tripartite α‑pore‑forming toxin and a major *Bacillus cereus* virulence factor. Although LITAF and its homolog CDIP1 were previously identified as host receptors that promote HBL‑induced cytolysis, mice lacking both proteins remain only partially protected, indicating the existence of additional determinants of susceptibility. Using genome‑wide CRISPR knockout screens in *Litaf*^*−/−*^/*Cdip1*^−/−^ mouse embryonic fibroblasts and human HT1080 cells, we identify caveolin‑1 (CAV1), a caveola‑associated membrane protein, as a third conserved host factor required for HBL cytolytic activity. Confocal imaging and co‑immunoprecipitation analyses show that CAV1 colocalizes with HBL and physically associates with the toxin at the plasma membrane. Notably, mice lacking all three factors (Litaf/Cdip1/Cav1 triple knockouts) are completely resistant to HBL toxin challenge, establishing CAV1 as an additional host factor mediating HBL toxin cytotoxicity. These findings define a tripartite host factor system required for HBL toxin activity and position CAV1 as an additional host determinant to *B. cereus* virulence.

## Author summary

Hemolysin BL (HBL), a tripartite pore‑forming toxin produced by *Bacillus cereus*, relies on host receptors to trigger cytolysis. Although HBL toxin receptors LITAF and CDIP1 promote susceptibility to HBL, their loss provides only partial protection in mice, suggesting additional host determinants. Genome‑wide CRISPR screens in LITAF/CDIP1‑deficient mouse and human cells identify caveolin‑1 (CAV1) as a conserved, additional host factor required for HBL toxin‑mediated cytotoxicity. CAV1 physically associates with HBL at the plasma membrane, and mice lacking all three receptors/factors (LITAF, CDIP1, and CAV1) are completely resistant to HBL challenge. These findings define a tripartite receptor/factor system essential for HBL toxicity and reveal CAV1 as a key host determinant of *B. cereus* virulence.

## Abstract 3

Bacillus cereus hemolysin BL (HBL) uses the host receptors LITAF and CDIP1 for cytotoxicity, but the loss of both only protects the host partly. This study shows that Caveolin 1 completes a three-factor host system whose combined loss confers full resistance to HBL.

## Introduction

*Bacillus cereus* is a toxin-producing, facultatively anaerobic, gram-positive pathogenic bacterium commonly found in soil and food products. It is capable of causing severe food poisoning and life-threatening infections through toxin production [1]. *B. cereus* induces two types of gastrointestinal illness: emetic (vomiting) and diarrheal [2,3]. The emetic form is characterized by nausea, vomiting, and abdominal cramps, resulting from ingestion of food contaminated with *B. cereus* toxins [2,3]. In contrast, the diarrheal form occurs when enterotoxins produced by *B. cereus* inside the host affect the intestinal mucosa [2,3]. Beyond foodborne illness, *B. cereus* has increasingly been recognized as a significant pathogen responsible for acute severe infections and fatalities in immunocompromised patients and children [4–9]. Among its virulence factors, *B. cereus* produces two distinct tripartite pore-forming toxins (PFTs): hemolysin BL (HBL) and nonhemolytic enterotoxin [3,10–14].

PFTs are a major class of virulence factors found in numerous bacterial pathogens, accounting for ~30% of all known protein toxins [15,16]. Studies have shown that *B. cereus* strains deficient in HBL toxin exhibit significantly reduced virulence in mice, highlighting HBL as a key virulence factor of *B. cereus* [17,18]. PFTs induce cellular damage by creating pores in the plasma membrane, thereby compromising the integrity of target mammalian cells. Based on the secondary structures of their membrane-spanning components, PFTs are classified into two types: α-PFTs, which form pores using a ring of amphipathic α-helices; β-PFTs, which employ a β-barrel structure to penetrate the membrane [16,19]. Many β-PFTs utilize plasma membrane cholesterol as a cellular receptor, facilitating binding and cytolytic activity. Due to this mechanism, they are categorized as cholesterol-dependent cytolysins (CDCs) [20,21].

HBL toxin is an α-pore-forming toxin (α-PFT) composed of three distinct protein components: the cellular binding B subunit (HBL-B, 42.5 kDa) and two cytolytic subunits, L1 (HBL-L1, 43.8 kDa) and L2 (HBL-L2, 49.3 kDa) [11,22]. While individually nontoxic, these components sequentially assemble on the plasma membrane surface, first HBL-B, followed by HBL-L1, and finally HBL-L2, to form cytolytic pores that disrupt membrane integrity [11,22]. Notably, HBL toxin and certain other PFTs have evolved to utilize specific cellular proteins as receptors [18,23–25], enhancing their ability to bind and exert their cytolytic effects. HBL-B, the cellular binding moiety of HBL toxin, initiates cytolytic action by binding to its cellular protein receptors. This binding triggers the sequential recruitment of HBL-L1 and HBL-L2, culminating in the formation of a membrane-penetrating pore structure that leads to cell death [11,22]. Previously, we identified **L**PS-**i**nduced **T**NF-**α f**actor (LITAF) and its related protein CDIP1 (**c**ell **d**eath **i**nvolved **p**53 target 1, also called LITAF-like) as alternative cellular receptors facilitating HBL toxin binding on target cells [18]. Consequently, the deletion of both LITAF and CDIP1 is necessary for B16-F10 cells to achieve complete resistance to HBL toxin-induced cytolysis.

To explore the in vivo roles of LITAF and CDIP1 in HBL pathogenesis, here we generated and characterized *Cdip1*^−/−^ mice, as well as combined *Litaf*^−/−^/*Cdip1*^−/−^ mice. Unexpectedly, although *Litaf*^−/−^/*Cdip1*^−/−^ mice showed increased resistance to HBL toxin exposure, they ultimately succumbed following repeated dosing. These findings suggest that an additional receptor may contribute to HBL’s in vivo toxicity.

To identify this additional receptor, we performed genome‑wide CRISPR knockout screens in two cell systems lacking LITAF and CDIP1: immortalized mouse embryonic fibroblasts (iMEFs) derived from *Litaf*^*−/−*^*/Cdip1*^*−/−*^ mice and human HT1080 cells deficient in both genes. These unbiased screens revealed Caveolin‑1 (CAV1), a caveola‑associated membrane protein, as an HBL receptor that mediates the toxin’s cytolytic activity in the absence of LITAF and CDIP1. Mutagenesis analyses further showed that HBL‑B engages CAV1 embedded in the cytosolic leaflet of the plasma membrane via its transmembrane‑competent β‑tongue (β‑hairpin) structure. Confocal microscopy and co‑immunoprecipitation assays confirmed the colocalization and physical association of cell‑bound HBL‑B with CAV1. Whereas *Litaf*^*−/−*^*/Cdip1*^*−/−*^ mice displayed partial resistance to HBL, triple‑knockout *Litaf*^*−/−*^*/Cdip1*^*−/−*^*/Cav1*^*−/−*^ mice were completely resistant, demonstrating the physiological importance of CAV1 in HBL pathogenesis. Together, these findings establish a tripartite receptor/factor system required for HBL toxicity and identify CAV1 as an additional host determinant of *B. cereus* HBL toxin virulence.

## Results

### An additional host factor mediating cytotoxicity of HBL toxin

**Fig 1:**
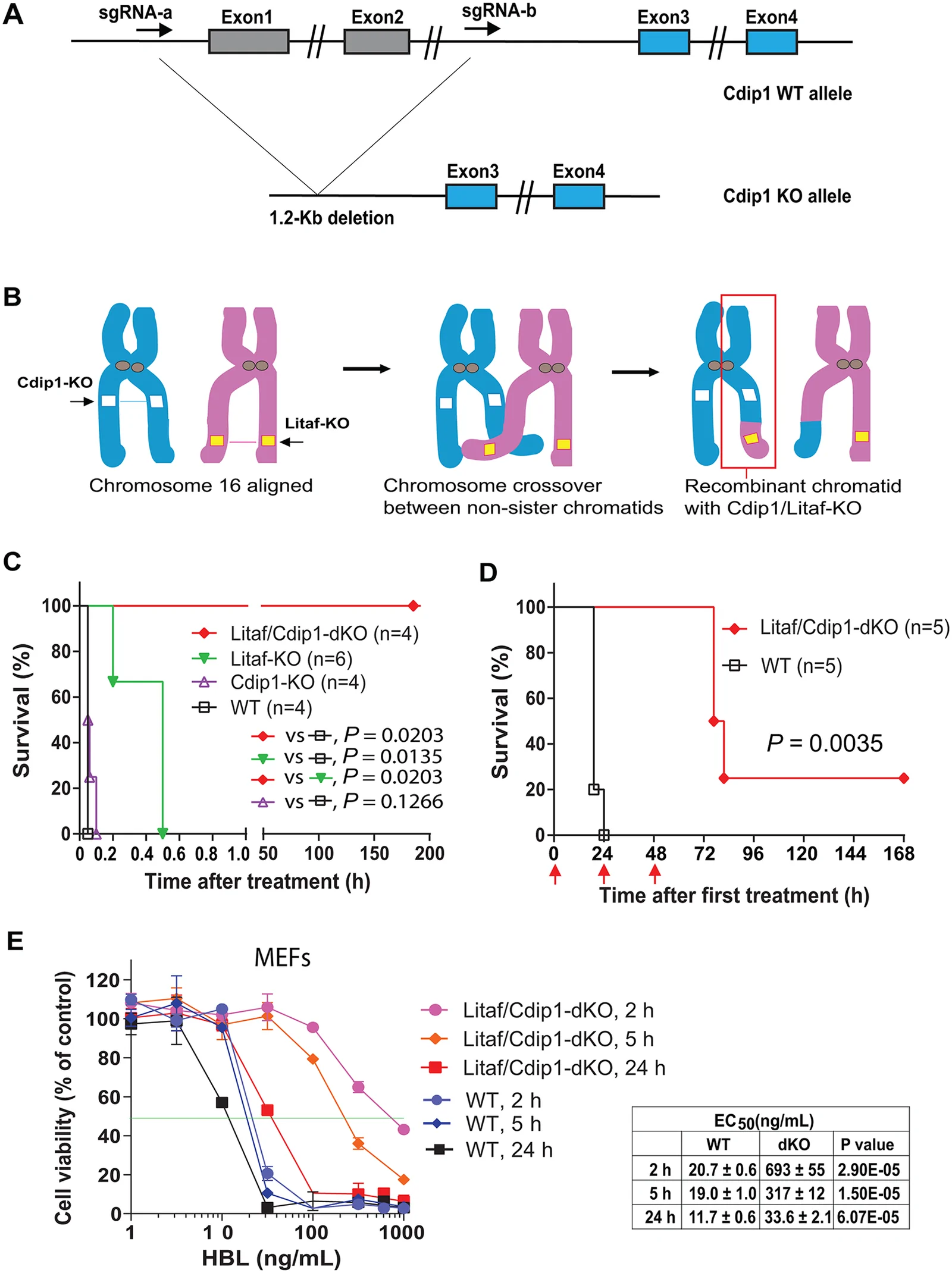
In vivo roles of LITAF and CDIP1 in HBL toxin pathogenesis. **(A)** Generation of *Cdip1*-null mice. The four exons of the mouse *Cdip1* gene and the two sgRNAs used for generation of *Cdip1* mutant mouse are shown. A mutant mouse line containing a deletion of 1.2-Kb *Cdip1* gene fragment mediated by the two sgRNAs was obtained. This deletion results in loss of the first two exons including the start codon, leaving the remaining exons 3 and 4 out of the protein-coding frame. Thus, this mouse line is defined as *Cdip1*-KO. **(B)** Schematic representation of chromosome 16 crossover during meiosis. Exchange of chromosome segments between nonsister chromatids in meiosis resulted in recombinant chromatid, yielding *Litaf/Cdip1* double-KO allele, which was used for the subsequent breeding for generation of *Litaf/Cdip1*-double KO mice. **(C)** Mice with single *Cdip1*, *Litaf* or their double KO mice were challenged with 2 µg HBL toxin (defined as 2 µg each of HBL-B, HBL-L1, and HBL-L2) (I.V.). Log-rank test, *P < 0.01*, between *Litaf/Cdip1*-dKO mice and other groups. Of note, *Litaf/Cdip1*-dKO mice survived 1 x 2 µg HBL toxin challenge. Log-rank test. *P* values from pairwise comparisons were adjusted using the Holm procedure to control the family-wise error rate. Adjusted *P* values were used to determine statistical significance (*P* < 0.05). **(D)** WT and *Litaf/Cdip1* double KO mice were challenged with 2 µg HBL toxin (I.P. for multiple injections) as indicated by red arrows. Of note, *Litaf/Cdip1*-dKO mice succumbed to three doses (3x) of 2 µg HBL toxin challenge. Log-rank test. **(E)** MEF-WT and MEF (*Litaf/Cdip1*-dKO) cells were treated with various concentrations of HBL toxin for 2 h, 5 h, or 24 h, followed by an MTT assay to assess cell viability. MEF (*Litaf/Cdip1*-dKO) cells are only partially resistant to HBL toxin, implicating that a third functional receptor may exist in MEFs. EC_50_ values represent the toxin concentrations required to kill 50% of the cells. Means ± SD of three independent biological replicates. *P* values obtained using unpaired two-tailed Student *t* tes*t*. The numerical data underlying this figure can be found in S1 Data.

To investigate the in vivo roles of HBL toxin receptors in pathogenesis, we previously generated *Litaf*-KO mice [18], and have further generated *Cdip1*-KO mice in this study. The *Cdip1*-KO mouse line was generated using a CRISPR gene editing approach to delete a 1.2-Kb *Cdip1* genomic fragment containing *Cdip1*’s exons 1 and 2 including the start codon (Fig 1A). *Cdip1*^−/−^ mice were born with the expected mendelian ratio when *Cdip1*^+/−^ × *Cdip1*^+/−^ were crossed and survived to adulthood without apparent abnormality. We challenged *Cdip1*^*−/−*^ mice and their wildtype (WT) control mice with HBL toxin (1 μg) via intraperitoneal injection (I.P.) and found that the *Cdip1*^*−/−*^ mice were not significantly more resistant to a toxin challenge (S1 Fig). This was most likely due to the presence of the LITAF receptor in these mice that masked the potential role of CDIP1 in mediating the in vivo toxicity of HBL toxin. We then sought to generate *Litaf/Cdip1*-double KO mice. However, *Cdip1* gene (Chromosome 16, 2.47 cM) and *Litaf* gene (Chromosome 16, 6.28 cM) are located on the same chromosome 16 in the mouse genome within 3.81 cM (centimorgan) apart (Fig 1B). As such, generation of *Litaf/Cdip1*-double KO mice relied on the spontaneous homologous chromosome crossover event during mitosis that occur at much lower rates (Fig 1B). Fortunately, through a scaled-up breeding effort, we successfully obtained (*Litaf/Cdip1*)^+/−^ mice as breeding founders, and subsequently generated *Litaf/Cdip1*-double KO (*Litaf*^−/−^/*Cdip1*^−/−^) mice. *Litaf*^−/−^/*Cdip1*^−/−^ mice did not exhibit any obvious phenotypes, demonstrating that both *Litaf* and *Cdip1* are nonessential genes in mice. We then challenged *Litaf* and *Cdip1* single KO mice, their double KO mice, and their littermate WT control mice with HBL toxin via intravenous injection (I.V.). As expected, while all the WT mice and single KO mice succumbed to a single toxin injection of 2 μg HBL (defined as 2 μg each component), all *Litaf*^−/−^/*Cdip1*^−/−^ mice survived (Fig 1C). However, unexpectedly, *Litaf*^−/−^/*Cdip1*^−/−^ mice became susceptible to the toxin when challenged with three doses of 2 μg HBL toxin (I.P. route for multiple injections) which caused roughly 80% mortality (Fig 1D). We isolated mouse embryonic fibroblasts (MEFs) from WT and *Litaf*^−/−^/*Cdip1*^−/−^ mice. In cytotoxicity assays, while MEFs (WT) were sensitive to HBL toxin (Fig 1E), *Litaf*^−/−^/*Cdip1*^−/−^ MEFs were only partially (~10-fold) more resistant than WT MEFs to HBL toxin (Fig 1E). These results suggested that an additional HBL toxin receptor exists to mediate the toxicity of HBL toxin in *Litaf*^−/−^/*Cdip1*^−/−^ mice and in the MEFs in the absence of LITAF and CDIP1.

### CAV1 as an additional host factor mediated HBL cytotoxicity

**Fig 2:**
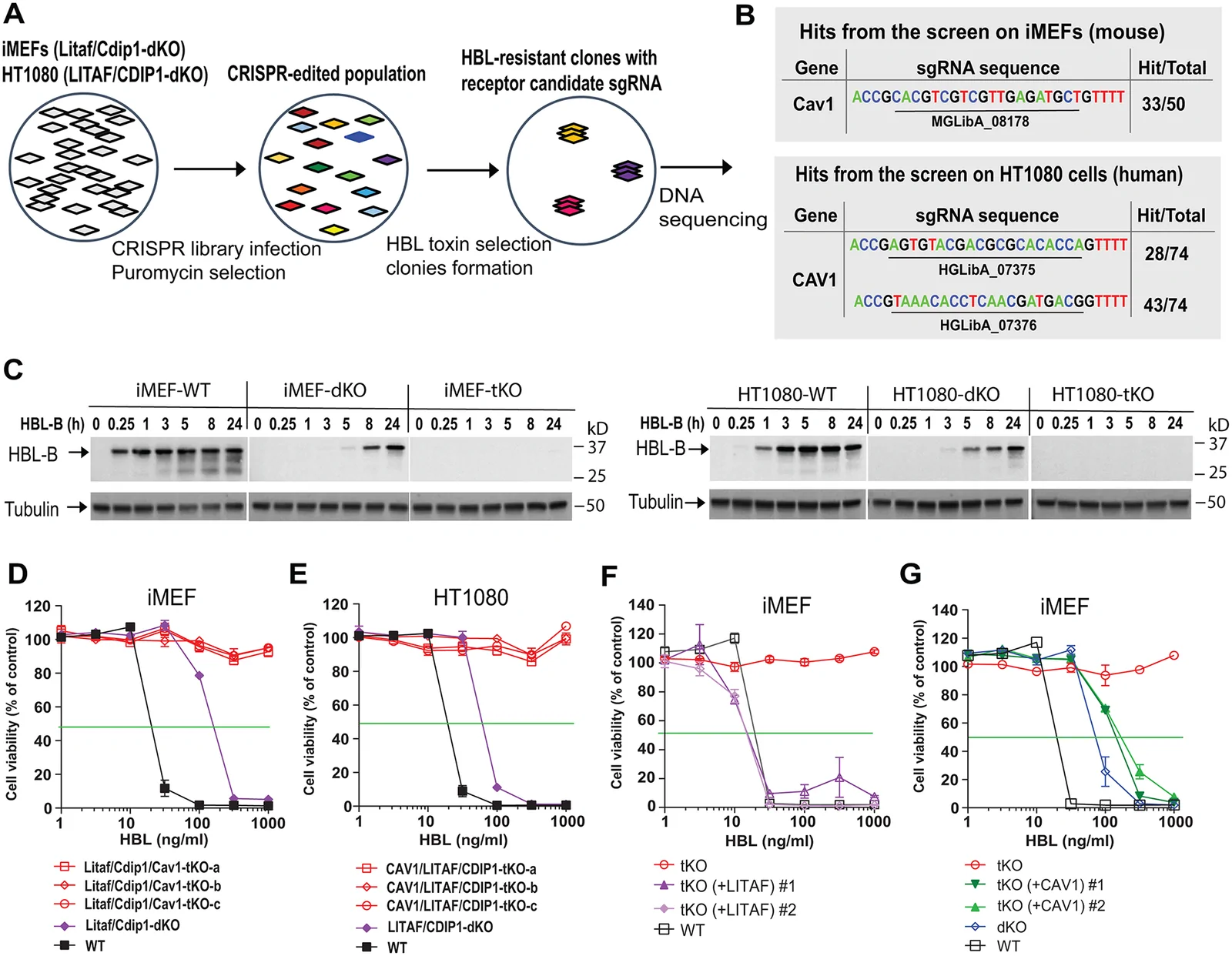
Whole-genome CRISPR KO screens identify CAV1 as an additional potential HBL toxin receptor. **(A)** iMEF-dKO cells infected with the mouse GeCKO library-A (after puromycin selection) were treated with 1 μg/mL HBL for 24 h. After 10 days, only HBL-resistant clones formed colonies and were subjected for DNA sequencing to identify the sgRNAs present in each clone. Similar CRISPR KO screen using the human GeCKO library-A was performed on HT1080 (LITAF/CDIP1-dKO) cells. **(B)** DNA sequencing of sgRNAs from HBL-resistant clones revealed that CAV1 is the only hit from both the mouse and human CRISPR KO screens. **(C)** CAV1 is required for HBL toxin’s cellular association in the absence of LITAF and CDIP1. iMEFs (WT, *Litaf/Cdip1*-dKO (dKO), and *Litaf/Cdip1/Cav1*-tKO (tKO)) and HT1080 (WT, dKO, and tKO) cells were incubated with HBL-B component (1 μg/mL) for various lengths of time at room temperature (23°C). After washing off unbound HBL-B, cell lysates were prepared and analyzed by Western blotting using rat anti-HBL-B antiserum or anti-tubulin antibody. Notably, while dKO cells retained partial HBL-B associating ability, no HBL-B association was observed in iMEF-tKO or HT1080-tKO cells. Representative of three independent experiments with similar results. **(D, E)** CAV1 is required for HBL toxin’s cytolytic activity to iMEFs and HT1080 cells lacking LITAF and CDIP1. iMEF-tKO cells (three independent tKO clones: a, b, c) (D) and HT1080-tKO cells (three independent tKO clones: a, b, c) (E) were generated by CRISPR KO of CAV1 in iMEF-dKO cells and HT1080-dKO cells, respectively. Cells were incubated with various concentrations of HBL toxin for 4 h, followed by an MTT assay to assess cell viability. Of note, iMEF-tKO cells and HT1080-tKO cells are completely resistant to HBL toxin. Data are represented as mean ± SD of at least three independent biological replicates. **(F, G)** Reconstitution of iMEF-tKO cells with LITAF or CAV1 restores the cells’ sensitivity to HBL toxin. iMEF-tKO cells transfected with either LITAF-expressing plasmid (F) (two clones #1 and #2) or CAV1-expressing plasmid (G) were treated with various concentrations of HBL toxin for 24 h, followed by an MTT assay. Mean ± SD of at least three independent biological replicates. The numerical data underlying this figure can be found in S1 Data.

We hypothesized that this additional toxin receptor could be identified by an unbiased approach, specifically a genome-wide CRISPR/Cas9 knockout (GeCKO) screen using MEFs (*Litaf*^−/−^/*Cdip1*^−/−^, or dKO) that lack LITAF and CDIP1. To do so, we first immortalized MEFs (*Litaf*^−/−^/*Cdip1*^−/−^) by infecting the cells with a recombinant lentivirus containing both large and small SV40 T antigens [26] (see Materials and methods), and then further transfected the cells with a Cas9-expressing plasmid, resulting in **i**mmortalized MEFs (*Litaf*^−/−^/*Cdip1*^−/−^, Cas9), termed iMEF-dKO, which were suitable for the subsequent CRISPR KO screen. We then conducted a GeCKO screen on these iMEF-dKO cells using the mouse GeCKO lentiviral pooled library-A [27,28], which targets 20,611 mouse genes with each gene targeted by three different single-guided RNAs (sgRNAs) (Fig 2A). We reasoned that the HBL-resistant cells isolated after infection with a GeCKO lentiviral pooled library and the subsequent toxin exposure should be enriched for cells in which the toxin receptor gene(s) had undergone sgRNA-mediated KO and its sgRNAs could be subsequently identified by DNA sequencing (Fig 2B).

Incubation of iMEF-dKO cells infected with the mouse GeCKO library-A with a lethal concentration of HBL toxin (1 µg/mL, defined as 1 µg/mL each of HBL-B, L1, L2 combined) for 24 h killed all sensitive cells, leaving a small number of surviving cells that grew to form ~100 HBL toxin-resistant clones (from eight different culture plates, representing at least eight independent events) (Fig 2A). To reveal identities of sgRNAs in each of these toxin-resistant iMEF-dKO clones, genomic DNA was extracted from each clone, and PCR fragments containing sgRNA sequences were prepared and subjected to Sanger DNA sequencing. Remarkably, among 50 clones that gave clear readable sgRNA sequences, 33 contained Cav1 sgRNA (MGLibA_08178, sgRNA ID in mouse GeCKO lentiviral library-A [27,28]) (Fig 2B). This Cav1 sgRNA (MGLibA_08178) was found in HBL-resistant clones from all the eight plates. These results indicated that CAV1 may function as the “additional host factor” mediating HBL toxin cytolytic activity in iMEF-dKO cells.

To further verify the above results, we performed an independent GeCKO screen on human HT1080 cells with both LITAF and CDIP1 knocked out (termed HT1080-dKO) using the human GeCKO lentiviral pooled library-A [27,28]. Intriguingly, among the 74 HBL-resistant clones that survived exposure to 1 µg/mL HBL toxin, CAV1 sgRNA (HGLibA_07375) was found in 28 clones and CAV1 sgRNA (HGLibA_07376) was found in 43 clones (Fig 2B). Thus, CAV1 gene was the only gene identified in both mouse and human GeCKO screens. These results demonstrate the species-independent role of CAV1 in mediating HBL cytolytic activity in the absence of LITAF and CDIP1.

### CAV1 in mediating HBL’s cellular binding and cytolytic activity

To investigate the role of CAV1 in HBL toxin pathogenesis in the absence of LITAF and CDIP1, we further knocked out *Cav1* in both *Litaf/Cdip1*-double KO iMEF (dKO) and HT1080 (dKO) cells using CRISPR gene editing, resulting in *Litaf/Cdip1/Cav1*-**t**riple KO cells, namely iMEF-tKO and HT1080-tKO cells. To determine whether CAV1 mediates the HBL toxin cellular binding in the absence of LITAF and CDIP1, we incubated HBL-B component (1 µg/mL), the cellular binding moiety of HBL toxin, with WT, *Litaf/Cdip1*-dKO, and *Litaf/Cdip1/Cav1*-tKO iMEFs and HT1080 cells for various lengths of time. While iMEF-dKO and HT1080-dKO cells retained HBL-B binding ability, albeit to a lesser extent than the WT cells, iMEF-tKO and HT1080-tKO cells completely lost HBL‑B association (Fig 2C).

In cytotoxicity assays, iMEF‑tKO and HT1080‑tKO cells were completely resistant to HBL toxin, whereas iMEF‑dKO and HT1080‑dKO cells remained susceptible, though less so than WT cells (Fig 2D and 2E). Thus, in the absence of LITAF and CDIP1, CAV1 is required not only for HBL toxin binding but also for its cytolytic activity. Reconstitution of iMEF‑tKO cells with either LITAF (tKO (+ LITAF)) or CAV1 (tKO (+ CAV1)) restored sensitivity to HBL toxin (Fig 2F and 2G), indicating that CAV1 functions as a potential receptor for HBL toxin.

In a control experiment, we confirmed that the HBL‑B component is absolutely required for the toxin’s cytolytic activity, even when CAV1 alone (in the absence of LITAF and CDIP1) is used as the potential toxin receptor. We showed that in the absence of HBL-B, HBL-L1 plus HBL-L2 failed to display cytolytic activity to iMEF-tKO (+ CAV1) and iMEF-dKO cells, which only express CAV1 (S2A Fig). Similarly, HBL-L1 plus HBL-L2 did not show cytotoxicity to HT1080-WT cells and HT1080-dKO cells (S2A Fig). We confirmed that each individual component of the HBL toxin is noncytotoxic, even at concentrations as high as 2 µg/mL following 24 h of incubation (S2B Fig).

To determine whether endocytosis is required for the cytolytic activity of HBL toxin, we pre‑treated WT and dKO iMEFs as well as HT1080 cells with bafilomycin A1 or ammonium chloride (NH_4_Cl) (S3A Fig), two agents that inhibit receptor‑mediated endocytosis by blocking endosomal acidification. As expected, both inhibitors markedly suppressed cytotoxicity induced by the modified anthrax toxin (PA + FP59) [29] (S3B Fig). In contrast, neither treatment altered HBL‑mediated cytolysis in these cells. (S3A Fig). Because endocytosis is a temperature‑sensitive and becomes negligible below 16°C [30,31], we next incubated HT1080 cells and iMEFs with HBL toxin or the modified anthrax toxin at 15°C, an endocytosis‑impermissive condition. Anthrax toxin‑mediated cytotoxicity was completely abolished at this temperature, whereas HBL‑induced cytolysis remained largely unaffected (S3C and S3D Fig). Together, these findings indicate that endocytosis is unlikely to play a role in the cytolytic mechanism of HBL toxin.

### CAV1 appears not to affect other HBL toxin receptors

Glycosylphosphatidylinositol (GPI)-anchored proteins, which attach to cell surface via GPI lipid anchor, are enriched in caveolae. GPI-anchored protein CD59 is a receptor for the cholesterol-dependent cytolysin intermedilysin, a β-PFT secreted by *Streptococcus intermedius* [23]. To rule out the possibility that certain GPI-anchored proteins regulated by CAV1 may directly mediate the HBL binding, we treated HT1080 WT cells and HT1080-dKO cells with Phosphatidylinositol-specific phospholipase C (PI-PLC), which can enzymatically remove GPI-anchored proteins from cell surface. While PI-PLC treatment could diminish GPI-anchored protein CD59, the same treatment could not affect HBL toxin binding to either WT or dKO cells (S4 Fig), suggesting that GPI-anchored proteins are unlikely to play roles in mediating the cellular binding of HBL toxin.

To test whether the role of CAV1 in mediating HBL’s cytolytic activity is through indirectly regulating localization of other HBL toxin receptors (LITAF, CDIP1, or other potential molecules enriched in caveolae) on the cell plasma membrane, we isolated primary lung endothelial cells (ECs) and BMDMs from *Cav1*^*−/−*^ and WT control mice. Notably, all these *Cav1*-KO cells remained similar sensitivity as the WT cells to HBL in cytotoxicity assays (S5 Fig), indicating that CAV1 does not affect other HBL toxin receptors.

### Colocalization of HBL-B with CAV1 on the cell surface

**Fig 3:**
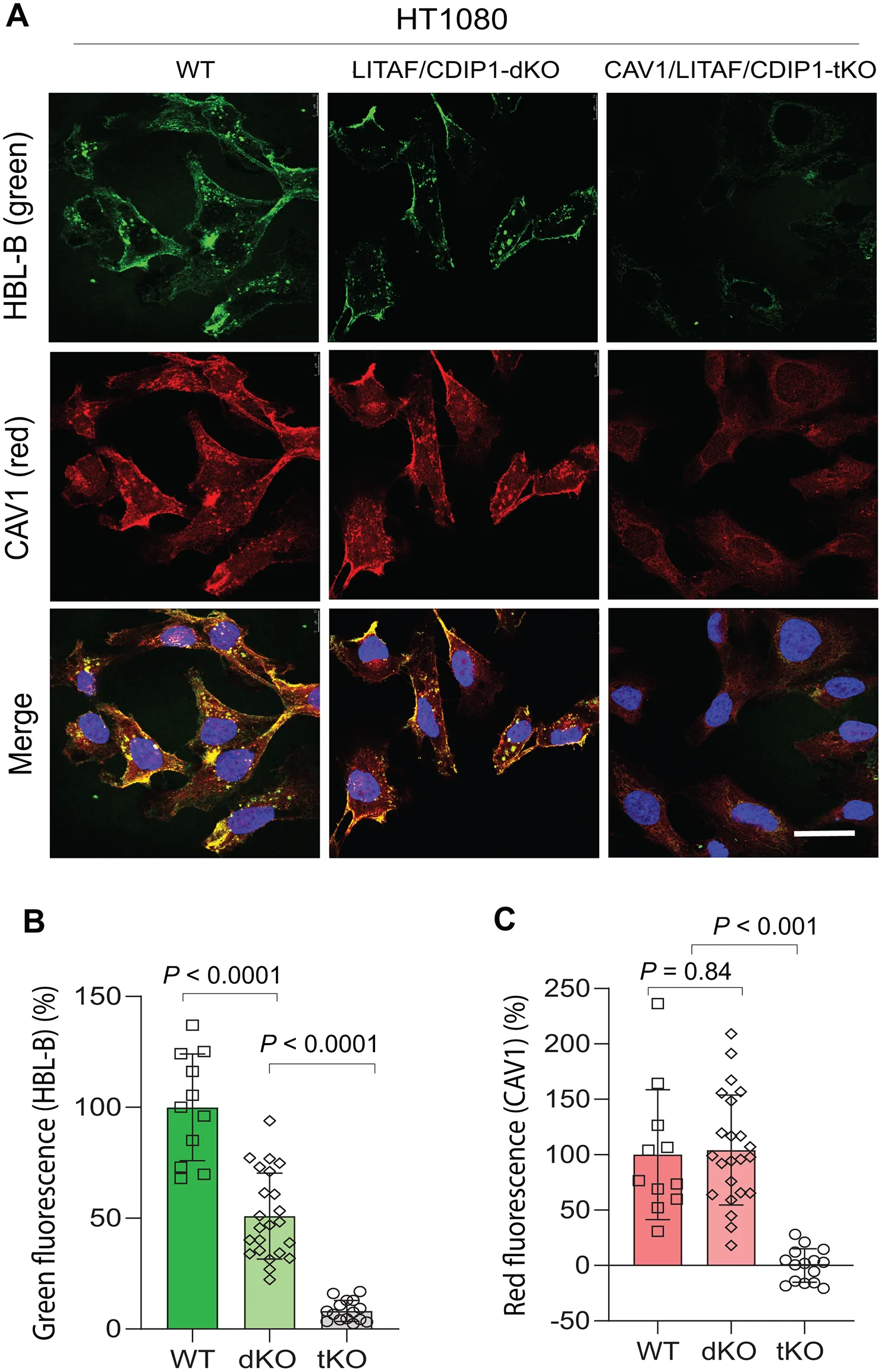
Co-localization of HBL-B and CAV1 on the cell surface. **(A)** Co-localization (yellow) of HBL-B (green) and CAV1 (red) on HT1080 cell surface. HT1080-WT, HT1080-dKO, and HT1080-tKO cells bound with HBL-B were stained with an anti-HBL-B antiserum (green) and an anti-CAV1 antibody (red). Merge images highlight co-localization (yellow) of HBL-B and CAV1. DAPI-stained nuclei (blue). Scale bar = 25 μm. Representative of three independent experiments with similar results. **(B)** Green fluorescence (HBL-B) intensities in HT1080-WT, -dKO, and -tKO cells. Each dot represents a single cell. Data are expressed as mean ± SD, relative to the average intensity of WT cells. **(C)** Red fluorescence (CAV1) intensities in HT1080-WT, -dKO, and -tKO cells. Each dot represents a single cell. After normalization to tKO cells, data are expressed as mean ± SD, relative to the average intensity of WT cells. The numerical data underlying this figure can be found in S1 Data.

The above results suggest that CAV1 might function as an additional HBL toxin receptor. As such, we reasoned that HBL toxin and CAV1 should co-localize on the cell surface. To examine this, we performed confocal microscopy analyses on WT, dKO, and tKO HT1080 cells bound with HBL-B. The cells were stained with an anti-HBL-B antiserum and an anti-CAV1 antibody (Fig 3). Three key observations were revealed: (1) Agreeing well with the above toxin cellular binding results (Fig 2C), HBL-B signal (green) decreased in HT1080-dKO cells compared to that of HT1080-WT cells and minimal signal was detected in HT1080-tKO cells (Fig 3A and 3B). This also validates the specificity of the anti-HBL-B antiserum used. (2) The CAV1 signal (red) was readily detected in the CAV1-expressing HT1080-WT and HT1080-dKO cells, but not in HT1080-tKO cells (Fig 3A and 3C), verifying the specificity of the anti-CAV1 antibody. (3) The merged HBL-B (green) and CAV1 (red) signals showed remarkable co-localization (yellow) of CAV1 and HBL-B, suggesting that these proteins have a potential to interact with each other on the target cell surface (Figs 3 and S6). Remarkable co-localization of CAV1 and HBL-B could also be detected in iMEFs (S7 Fig).

### The HBL‑B β‑tongue in mediating toxin-cellular binding

**Fig 4:**
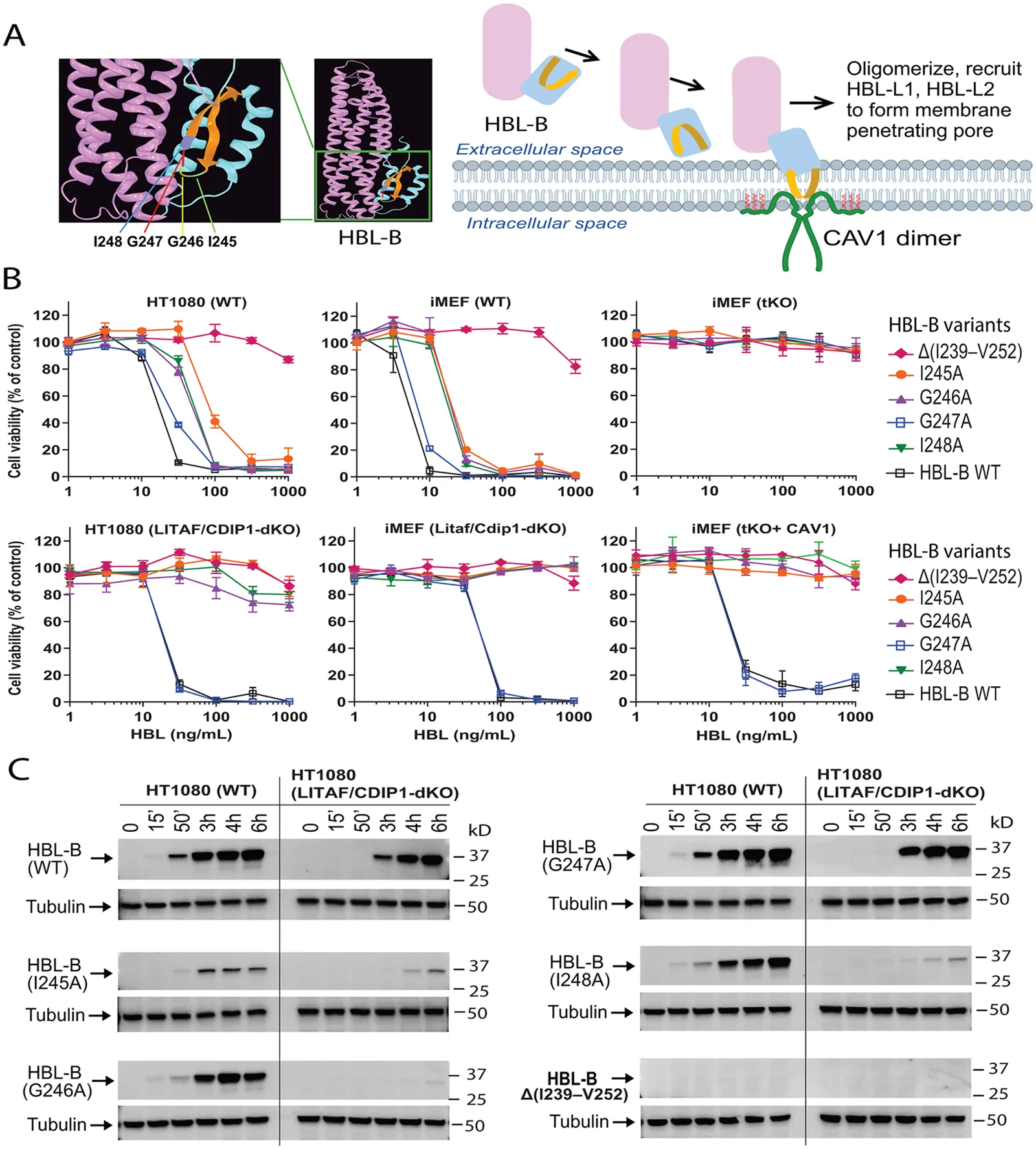
Role of the HBL‑B β‑tongue in cellular binding and cytolytic activity. **(A)** Structure of HBL‑B and proposed model for β‑tongue-mediated receptor engagement. The HBL‑B crystal structure (Left; Protein Data Bank accession 2NRJ, visualized using Protean 3D 18, DNASTAR Lasergene 18) is shown with the tail domain in pink and the head domain in cyan. The β‑tongue, composed of two antiparallel β‑strands (golden) within the head domain, is proposed to undergo a conformational rearrangement upon encountering the lipid bilayer. In the working model (Right; created in PowerPoint), the head domain turns away from the tail domain, allowing the hydrophobic β‑tongue to swing outward and partially insert into the membrane, thereby facilitating engagement with CAV1. **(B)** Cytolytic activity of HBL-L1, HBL-L2, plus each HBL-B variant to HT1080 and iMEF cells with indicated genotypes. HT1080 cells, MEFs, and iMEFs (tKO) reconstituted with or without CAV1 were treated with varying concentrations of HBL‑L1, HBL‑L2, and individual HBL‑B variants for 24 h. Cell viability was assessed by MTT assay. Deletion of the β‑tongue completely abolished cytolytic activity in both WT and LITAF/CDIP1 double‑knockout (dKO) cells. Point mutants HBL‑B(G246A), HBL‑B(I248A), and HBL‑B(I245A) retained partial activity in WT iMEFs but showed near‑complete loss of activity in CAV1‑expressing LITAF/CDIP1‑dKO HT1080 and iMEF cells. In contrast, HBL‑B(G247A) maintained activity comparable to WT HBL‑B. Data represent mean ± SD from at least three independent biological replicates. **(C)** Cellular association of HBL‑B variants. WT and dKO HT1080 cells were incubated with 1 µg/mL of each HBL‑B variant at 23°C for the indicated times. Cell lysates were analyzed by western blot using rat anti–HBL‑B antiserum or anti‑tubulin antibody. Consistent with its loss of cytolytic activity (B), the β‑tongue deletion mutant also lacked detectable cellular association. Point mutants HBL‑B (G246A), HBL‑B (I248A), and HBL‑B (I245A) showed only partial loss of association in WT cells but exhibited nearly complete loss of association in dKO cells. HBL‑B(G247A) retained cellular association comparable to WT HBL‑B. Representative results from three independent experiments. The numerical data underlying this figure can be found in S1 Data.

CAV1 is a small scaffolding protein that contributes to membrane curvature in structures such as caveolae and trafficking vesicles [32,33]. Because CAV1 is normally embedded in the inner leaflet of the plasma membrane, the binding component HBL‑B must first engage the plasma membrane to access CAV1. The crystal structure of HBL-B has a remarkable similarity with HlyE (PDB: 1QOY), an α-PFT from *E.coli* [34–36]. The crystal structure of HBL-B indicates that its β-tongue, a hydrophobic, transmembrane-competent β-hairpin composed of two anti-parallel β-sheets, is capable of inserting roughly halfway into the membrane lipid bilayer (Fig 4A) [36]. This raises the possibility that HBL‑B can reach and interact with CAV1 embed in the inner leaflet of the plasma membrane. Like CAV1, LITAF and CDIP1 are also classified as integral membrane proteins positioned at the cytosolic leaflet of the membrane [37,38], implying that HBL‑B must engage the membrane to access these receptors.

To investigate the role of the β‑tongue in HBL‑B–mediated cellular binding and cytolytic activity, we generated a panel of β‑tongue variants, including HBL‑B(I245A), HBL‑B(G246A), HBL‑B(G247A), and HBL‑B(I248A), in which each of the four residues near the tip of the β‑tongue was individually substituted with alanine. We also constructed a β‑tongue deletion mutant, HBL‑B Δ(I239–V252) (Figs 4A and S8). All variants were expressed in the BH500 host strain using pYS5‑based vectors (see Methods) and were secreted into the culture medium in their active form. Their expression and secretion levels were comparable to WT HBL‑B, with no detectable degradation, indicating that these mutations do not compromise protein stability (S8 Fig). Purified variants were then tested in cell‑binding and cytotoxicity assays.

The β‑tongue deletion mutant completely lost both cellular binding and cytolytic activity in WT and *LITAF*/*CDIP1*‑dKO HT1080 cells and iMEFs (Fig 4B and 4C), underscoring the essential role of the β‑tongue in membrane engagement. Interestingly, while the point mutants HBL‑B(I245A), HBL‑B(G246A), and HBL‑B(I248A) showed only partial reductions in binding and cytotoxicity in WT HT1080 and iMEF cells, these same variants exhibited a complete loss of activity in CAV1‑expressing *LITAF*/*CDIP1*‑dKO HT1080 and iMEF cells (Fig 4B and 4C). These findings indicate that residues I245, G246, and I248 are particularly important for receptor engagement, especially for interaction with CAV1. Consistent with this, *Litaf*/*Cdip1*/*Cav1*‑triple KO iMEFs reconstituted with CAV1 regained sensitivity to WT HBL‑B (in the presence of HBL‑L1 and HBL‑L2), but remained almost completely resistant to the I245A, G246A, and I248A variants (plus HBL‑L1 and HBL‑L2) (Fig 4B). In contrast, HBL‑B(G247A) retained near‑WT activity (Fig 4B and 4C). Together, these results demonstrate that the β‑tongue is essential for HBL‑B’s interaction with its cellular receptors and for mediating cytolytic activity. While residues near the tip of the β‑tongue (I245, G246, and I248) are especially critical for engagement with CAV1‑expressing cells (dKO or tKO + CAV1), alterations at these positions only partially impair interactions with LITAF/CDIP1‑expressing WT cells. This suggests that although the β‑tongue is required for membrane engagement, distinct residues may mediate interactions with CAV1 versus LITAF/CDIP1.

The C-terminal tail of CAV1 contains three palmitoylated Cysteine residues (C^133^, C^143^, and C^156^) [39,40]. Palmitoylation at these sites promotes the recruitment of CAV1 to detergent‑resistant membrane (DRM) domains such as caveolae. To test whether membrane localization is required for CAV1‑mediated HBL cytotoxicity, we generated a palmitoylation‑deficient CAV1 variant in which all three cysteines were substituted with alanine (C133A/C143A/C156A; CAV1‑3C). iMEF‑tKO cells were reconstituted with either CAV1‑3C or wild‑type CAV1. As expected, CAV1‑WT localized predominantly to DRMs, whereas CAV1‑3C showed markedly reduced DRM association (S9A and S9B Fig). Notably, while CAV1‑WT restored HBL sensitivity in iMEF‑tKO cells, those expressing CAV1‑3C remained fully resistant (S9C Fig), demonstrating that DRM localization is essential for CAV1 to mediate cytolytic activity of HBL toxin.

### Physical association of cell-bound HBL-B and CAV1

**Fig 5:**
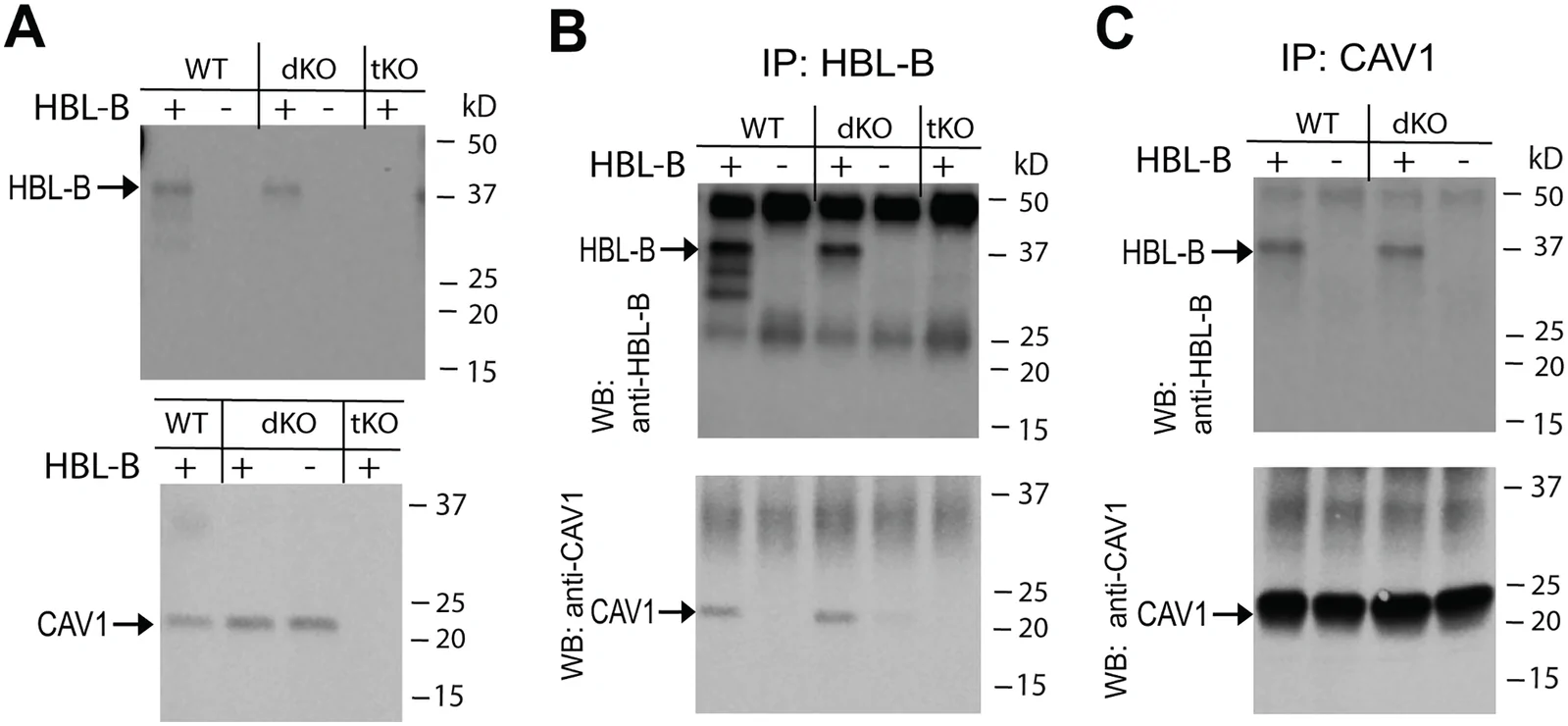
Co-immunoprecipitation of HBL-B with CAV1. **(A)** iMEFs with indicated genotypes (WT, *Litaf/Cdip1*-dKO, and *Litaf/Cdip1/Cav1*-tKO) were incubated with or without HBL-B (1 µg/mL) for 4 h at 23°C. Cells were washed twice to remove unbound HBL-B, and cell lysates were prepared in Triton X-100 lysis buffer at room temperature and analyzed by Western blotting using a rat anti-HBL-B anti-serum or anti-CAV1 antibody. Of note, *Litaf*/*Cdip1*/*Cav1*-tKO MEF cells lack expression of CAV1 and cannot bind HBL-B. Representative of three independent experiments with similar results. **(B, C)** Cell lysates prepared in (A) were immunoprecipitated using a rat anti-HBL-B antiserum (B) or a rabbit anti-CAV1 antibody (C). Then the immunoprecipitated samples were analyzed by Western blotting using the HBL-B antiserum and anti-CAV1 antibody. Of note, while HBL-B could not bind iMEF (tKO) cells, co-immunoprecipitation of HBL-B and CAV1 could be detected in both iMEF-WT as well as iMEF-dKO cells that express CAV1. Shown is a representative of three independent experiments with similar results.

To investigate whether HBL toxin and CAV1 physically interact, we performed co-immunoprecipitation (co-IP) assay. After incubation with HBL-B, the cell lysates of iMEF-WT, iMEF-dKO, and iMEF-tKO cells were immunoprecipitated by a rat anti-HBL-B antiserum or by an anti-CAV1 antibody. The immunoprecipitated samples were then analyzed by Western blotting using both the anti-CAV1 antibody and anti-HBL-B antiserum (Figs 5 and S10A). Interestingly, while HBL-B could not bind iMEF-tKO cells, HBL-B and CAV1 could be co-immunoprecipitated in both iMEF-WT and iMEF-dKO (Figs 5 and S10A). These results demonstrate that HBL-B and CAV1 are physically associated on the cell surface. We also confirmed that HBL-B could also co-immunoprecipitate, respectively, with CDIP1 and LITAF, verifying the physical association of HBL-B and these receptors (S10B and S10C Fig). Supporting the idea that HBL‑B–receptor interactions occur within the plasma membrane, co‑immunoprecipitation of HBL‑B with CAV1, LITAF, or CDIP1 was not increased by DTSSP (S10 Fig), a water‑soluble but membrane‑impermeable crosslinker that typically stabilizes associations between interacting cell‑surface proteins.

To assess potential direct interactions, purified HBL-B or its β‑tongue deletion variant and CAV1, either individually or in combination, were incubated in PBS or 0.1% DDM (n-dodecyl-β-D-maltoside, a mild detergent commonly used to solubilize membrane proteins while preserving their native structures [33, 41]) at room temperature for 30 min. The mixtures were subsequently resolved on a native gel to visualize potential protein complex formation by Western blotting using anti-CAV1 antibody and rat anti-HBL-B antiserum (S11 Fig). In PBS, CAV1 primarily existed as large aggregates that remained in the loading wells of the native gel. In contrast, CAV1 in DDM migrated into the gel as smaller oligomeric forms. In native gels, the migration rates of protein complexes are influenced not only by their size, but also by their conformation and net surface charge. HBL-B appeared predominantly as high-molecular-weight oligomers under both PBS and 0.1% DDM conditions. Notably, HBL-B and CAV1 co-migrated in DDM, indicating the formation of a distinct protein complex (S11 Fig). As expected, HBL-B β‑tongue deletion variant, which migrated much faster than WT HBL-B, could not form a complex with CAV1 (S11 Fig).

### In vivo role of CAV1 in HBL pathogenesis

**Fig 6:**
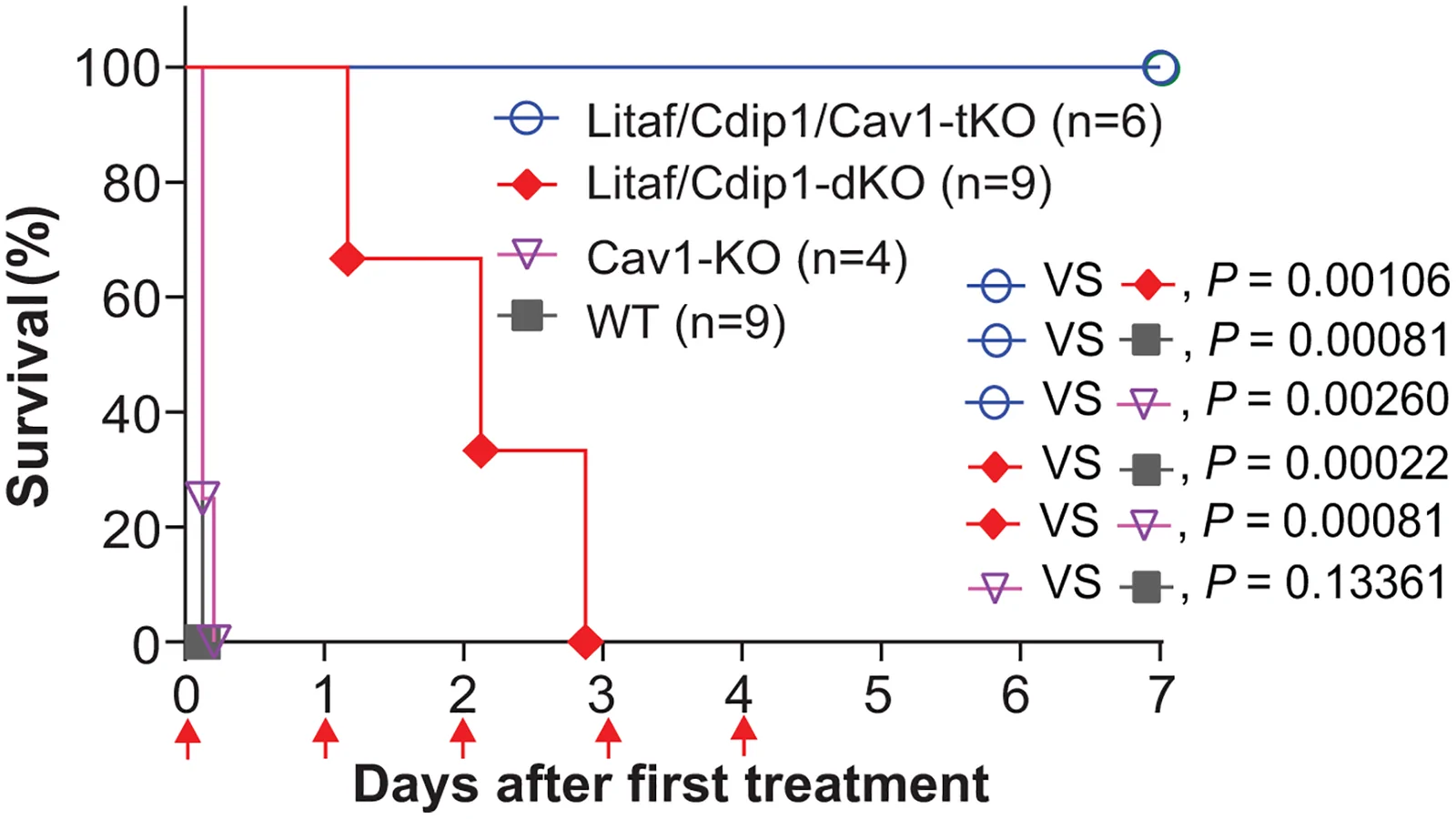
In vivo role of CAV1 in HBL pathogenesis. Mice with indicated genotypes (WT, *Cav1*^−/−^, *Litaf*^−/−^/*Cdip1*^−/−^ (dKO), *Litaf*^−/−^/*Cdip1*^−/−^/*Cav1*^−/−^ (tKO)) were challenged with 2 µg HBL toxin (I.P. route) daily as indicated by red arrows. Of note, while all the *Litaf*^*−/−*^*/Cdip1*^*−/−*^ (dKO) mice succumbed to 2-3 doses of HBL, the *Litaf*^−/−^/*Cdip1*^−/−^/*Cav1*^−/−^ (tKO) mice were completely resistant to the HBL challenge that all the mice survived even with 5 doses of 2 µg HBL toxin challenge. Log-rank test. *P* values from pairwise comparisons were adjusted using the Holm procedure to control the family-wise error rate. Adjusted *P* values were used to determine statistical significance (*P* < 0.05). The numerical data underlying this figure can be found in S1 Data.

To test whether the susceptibility of *Litaf*^*−/−*^*/Cdip1*^*−/−*^ mice to multiple doses of HBL is mediated by CAV1, we further generated *Litaf*^*−/−*^*/Cdip1*^*−/−*^/*Cav1*^*−/−*^ (tKO) mice by crossing *Litaf*^*−/−*^*/Cdip1*^*−/−*^ mice and *Cav1*^*−/−*^ mice. Strikingly, while WT and *Cav1*^*−/−*^ mice were killed by one dose of HBL and all the *Litaf*^*−/−*^*/Cdip1*^*−/−*^ mice succumbed to 2–3 doses of HBL, the resulted tKO mice were completely resistant to the HBL challenge that all the mice survived the challenge even with 5 doses of 2 μg HBL toxin (Fig 6). These results demonstrate the physiologically relevant role of CAV1 in HBL pathogenesis. These findings define three potential HBL toxin receptors in mediating the toxin in vivo toxicity and reveal CAV1 as a physically relevant host determinant of *B. cereus* HBL toxin virulence.

## Discussion

Whole‑genome CRISPR knockout screens have been instrumental in dissecting complex biological pathways and identifying host factors required for the action of bacterial toxins [18,28,42]. In this study, we performed two independent, saturating, and unbiased genome‑wide CRISPR KO screens in both mouse and human cells to identify additional host factors, beyond LITAF and CDIP1, that mediate the cellular binding and cytolytic activity of the HBL toxin. Remarkably, both screens converged on CAV1 as the sole hit. Loss of CAV1 in LITAF/CDIP1‑deficient cells completely abolished HBL binding and cytotoxicity, whereas reconstitution with CAV1 restored toxin sensitivity. Quantitative confocal microscopy and co‑immunoprecipitation further confirmed co‑localization and physical interaction between HBL‑B and CAV1 at the cell surface. Together, these findings establish CAV1 as a host factor (possibly the third HBL cellular receptor), acting alongside LITAF and CDIP1 to mediate toxin binding and cytolytic activity.

CAV1 is an essential component of caveolae, which are enriched in GPI‑anchored proteins. CAV1 is embedded in the inner leaflet of the plasma membrane, where it contributes to membrane curvature in caveolae and trafficking vesicles [32,33]. LITAF and CDIP1 are similarly small integral membrane proteins localized to the inner leaflet without obvious extracellular exposure. Thus, the HBL‑B binding component must engage the plasma membrane to access these receptors. The β‑tongue structure of HBL‑B has been proposed to insert into the lipid bilayer [36], suggesting a mechanism by which HBL‑B could reach receptors located on the inner leaflet. Supporting this model, our mutagenesis analyses demonstrated that β‑tongue variants of HBL‑B exhibit defects in both cellular binding and cytolytic activity. HBL-B variant with the β‑tongue deleted completely lost the toxin’s cellular binding and cytolytic activity to the cells that express all these potential receptors LITAF, CDIP1, and CAV1, indicating that the β‑tongue is an essential structure to engage with all these toxin receptors. Interestingly, while the residues near the tip of the β‑tongue, including I245, G246, and G248, are particularly critical for interaction with CAV1, alterations of these residues only partially affect the interactions with LITAF/CDIP1, suggesting that while the β‑tongue is essential in engagement with plasma membrane, it may use distinct key residues to interact with CAV1, LITAF, and CDIP1. Agreeing well with the notion that the interactions between HBL-B and its potential receptors occur within plasma membrane, the co-IP of HBL-B with CAV1, LITAF, and CDIP1 could not be enhanced by DTSSP, a water‑soluble but membrane‑impermeable crosslinker that typically stabilizes associations between interacting cell‑surface proteins.

Two other extracellular PFTs, staphylococcal α‑hemolysin and Clostridium perfringens ε‑toxin, have been shown to directly interact with CAV1 located at the inner leaflet of the plasma membrane [43,44]. Preincubation with recombinant CAV1 efficiently blocks the hemolytic activity of α‑hemolysin in a dose‑dependent manner [43]. In addition, ε‑toxin binds to and forms complexes with CAV1 or CAV2, an interaction that is particularly important for ε‑toxin oligomerization and subsequent pore formation at the plasma membrane [44].

GPI‑anchored proteins serve as receptors for intermedilysin, a β‑PFT secreted by *Streptococcus intermedius* [23]. However, removal of GPI‑anchored proteins by PI‑PLC treatment did not affect HBL binding, ruling out the possibility that CAV1 contributes to HBL pathogenesis by regulating GPI‑anchored protein abundance. Moreover, *Cav1*‑KO cells and *Cav1*‑KO mice remain susceptible to HBL toxin unless LITAF and CDIP1 are also absent, indicating that CAV1 does not regulate the membrane localization of the other two receptors.

These three potential HBL receptors, LITAF, CDIP1, and CAV1, have each been implicated in innate immune responses or in cellular defense against plasma membrane damage caused by pore‑forming toxins. LITAF (LPS‑Induced TNF‑α Factor) was originally identified for its role in upregulating TNF‑α during microbial infection [45–48]. CDIP1, although less well characterized, is upregulated under various cellular stress conditions [49]. LITAF was recently shown to protect against pore‑forming toxin‑induced cell death by promoting membrane repair [50]. In this study, a forward genetic screen was performed to identify mechanisms of host cell resistance to PFTs, and LITAF was identified as a mediator of protection from archetypal PFT *Staphylococcus aureus* α-toxin-induced cell death. Mechanistically, LITAF-mediated membrane repair proceeds through sequestration of α-toxin in intracellular multivesicular vesicles, followed by secretion as exosomes. This mechanism is activated by membrane flux, via the ubiquitin ligase NEDD4, and is effective against other PFTs and the endogenous pore GSDMD. Hence, LITAF is a mediator of membrane repair, linking damage sensing to membrane reorganization and turnover. CAV1 has also been implicated in membrane repair responses [51]. Thus, these observations suggest that *B. cereus* may have evolved to exploit host proteins that are induced during infection or membrane stress, thereby enhancing HBL’s cytolytic activity while simultaneously subverting host membrane repair mechanisms.

The facts that CAV1, LITAF, and CDIP1 are also present in trafficking‑vesicle membranes, and that many bacterial protein toxins (e.g., anthrax toxin) must be translocated into the target cell to exert cytotoxicity, raise the possibility that these proteins could function as trafficking machinery important for HBL toxin delivery into the cell. Although we cannot formally rule out this model, it is highly unlikely because of the following observations: (1) Like other pore‑forming toxins, HBL induces plasma membrane blebbing within minutes [18], indicating that the plasma membrane is its primary target. (2) Receptor‑mediated toxin internalization and trafficking are temperature‑sensitive processes that are largely inhibited between 4 and 16°C; under these conditions, anthrax toxin fails to intoxicate cells. In contrast, HBL remains fully active in plasma membrane pore formation at low temperatures (S3C Fig), demonstrating that its cytolytic activity is temperature‑insensitive and therefore unlikely to depend on endocytic trafficking.

*B. cereus* is an increasingly important human pathogen that causes food poisoning and severe infections through the secretion of exotoxins such as the pore‑forming HBL toxin. Many PFTs use plasma membrane cholesterol as their receptor; however, HBL has evolved to use the host proteins LITAF, CDIP1, and CAV1 to engage target cells. This receptor‑based mechanism enables HBL to bind efficiently to the plasma membrane, with EC_50_ values < 1 nM [18]. Notably, knockout of all three receptors is required to render both MEFs and mice completely resistant to HBL toxin, underscoring the physiological relevance of these host proteins in HBL pathogenesis. Reconstitution of triple‑KO cells with any one of the three receptors restored toxin sensitivity, demonstrating that each protein can function independently as an HBL receptor. All primary cells and cell lines examined to date are susceptible to HBL toxin [18], indicating that they express at least one member of this receptor set. This lack of strict cellular specificity, consistent with the broad expression of LITAF, CDIP1, and CAV1 (The Human Protein Atlas), is not unusual among pore‑forming toxins, which constitute one of the largest and most potent classes of bacterial virulence factors. Although it remains unclear why *B. cereus* evolved a toxin with such broad activity, its non‑selective cytolytic capacity aligns with the clinical manifestations observed once the bacteria breach local barriers. During systemic infection or bacteremia, HBL’s ability to damage diverse host cell types, including epithelial, endothelial, and immune cells, would be expected to accelerate tissue injury, promote vascular leakage, and compromise host defense mechanisms. These combined effects likely contribute to the rapid progression and severity of *B. cereus* infections in extra‑intestinal settings [4–9].

In summary, we demonstrate that the HBL toxin is a unique three‑component PFT that utilizes CAV1, LITAF, and CDIP1 as its potential cellular receptors. Mice deficient in all three factors exhibit complete resistance to repeated HBL toxin challenges, indicating that these three proteins constitute the full complement of host factors essential for *B. cereus* HBL‑mediated cytolysis.

## Materials and methods

### Study approval

All animal studies were carried out in a pathogen-free facility maintaining full accreditation by the American Association for the Accreditation of Laboratory Animal Care (AAALAC) and in accordance with protocols approved by the University of Pittsburgh Institutional Animal Care and Use Committee (Protocols: #22030855, #25036362). *Cav1* mutant mice with C57BL/6 background were obtained from Jackson Laboratory (#007083). *Litaf* KO (with 5.6-Kb DNA deletion in *Litaf* gene) mice with C57BL/6 background were generated previously [18]. *Cdip1* mutant mice with C57BL/6 background were made by CRISPR gene editing in this study.

### Proteins and protein purification

The HBL toxin components, HBL-B, HBL-L1, and HBL-L2 were expressed using pYS5-based expressing vector and purified from a nonvirulent *B. anthracis* strain BH500 as described [11]. BH500, which lacks all pathogenic plasmids, was featured with deletions of a sporulation gene and genes coding 10 different proteases [52]. Thus, this host strain is a suitable host (less protease activity) for making recombinant protein toxins. Using an expression vector containing anthrax protective antigen (PA) promoter and PA signal peptide allow secretion of the recombinant proteins into culture medium [53,54]. Recombinant proteins are usually secreted into culture medium with high yields in their active form, often reaching >100 mg per liter culture medium. HBL toxin components used in our work were directly purified from culture medium of host BH500 bacteria. Thus, two steps of chromatography (Q-Sepharose Fast Flow and Sephacryl S-200 high resolution gel filtration) we used can readily purify recombinant proteins to high purity with one prominent band at expected molecular mass.

HBL-B β-tongue variants were constructed by in-fusion cloning. These HBL-B variant proteins were expressed and purified as described for HBL-B above.

### *Litaf*, *Cdip1*, and *Cav1* knockout mice

*Cdip1* KO mouse line was generated using the CRISPR/Cas9 method [55,56]. Two CRISPR sgRNAs were designed and made using T7 in vitro transcription to target its exons. The sgRNA-a (TAGAGCATCCCAAGTGTCAG) and sgRNA-b (AAAAGCTTTGTATGCATAAG) targeted sequences upstream of the start codon and downstream of the 2nd exon, respectively, to mediate 1.2-Kb *Cdip1* gene fragment deletion. These sgRNAs were co-microinjected (20 ng/µl each sgRNA) with 50 ng/µl Cas9 mRNA (Trilink BioTechnologies) into fertilized eggs collected from C57BL/6N mice (Charles River). The injected embryos were cultured for 24 h in M16 medium (Millipore) and then implanted into the oviducts of pseudopregnant foster mothers. Mice born to the foster mothers were genotyped by PCR and DNA sequencing to identify founders with the desired mutation. We identified a mouse line with the desired 1.2-Kb deletion mediated by the two sgRNAs. This deletion results in loss of the first two exons including the start codon, leaving the remaining exons 3 and 4 out of the protein-coding frame. Thus, this mouse line is defined as *Cdip1*-KO.

Since the LITAF and CDIP1 genes are both located on mouse chromosome 16, substantial efforts were made to obtain the (*Litaf/Cdip1*)^±^ mice through rare, spontaneous chromosome crossover event during mitosis. We then successfully obtained *Litaf/Cdip1*-double KO (*Litaf*^−/−^/ *Cdip1*^−/−^) from crossing of (*Litaf/Cdip1*)^±^ mice. *Litaf*/*Cdip1*/*Cav1* triple-KO mice were generated by crossing *Litaf*^−/−^/*Cdip1*^−/−^ and *Cav1*^−/−^ mice.

### Whole-genome CRISPR knockout screen and CRISPR gene editing

The genome-wide CRISPR knockout lentiviral pooled libraries developed by Feng Zhang [27,28] were utilized for unbiased screens to identify potential host genes required for cytolytic activity of HBL toxin in the absence of LITAF and CDIP1. Both human and mouse versions of the CRISPR library-A were employed (#1000000049 (human), #1000000053 (mouse), Addgene). These libraries contain three independent single-guide RNAs (sgRNAs) for each of the 19,050 human genes and for each of the 20,611 mouse genes, respectively. As the libraries are in a two-vector system format, with Sp Cas9 encoded by a separate vector, we first transfected the lentiCas9-Blast plasmid (#52962, Addgene) [27,28] into iMEFs (*Litaf*^−/−^/*Cdip1*^−/−^) and HT1080 (LITAF/CDIP1-dKO) cells used for the screens. We followed the protocols as previously described [27,28] for the preparation and infection of the pooled lentiviral libraries. Briefly, the library DNA was packaged to form pooled lentiviral sgRNA libraries and titrated. For the mouse library-A screen, 30 million iMEFs (dKO) (Cas9-expressing) (500 × coverage of the library) were infected with the lentiviral pooled library A at a multiplicity of infection (MOI) of 0.3 [27,28]. Infected cells were then divided into eight 15-cm diameter culture dishes and selected with puromycin (5 μg/ml) for 3 days. Cells were passed by trypsinization to maintain 50% confluence for 1 week to allow completion of the gene editing process. Subsequently, for each cell plate, half of the cells were frozen for later genomic DNA isolation as the nonselected control, while the other half were cultured and subjected to HBL selection. Cells were incubated with 1,000 ng/mL HBL for 24 h, a concentration expected to kill all the iMEFs (dKO). Dead iMEFs dKO cells were removed by replacing the medium with fresh medium. Surviving cells were cultured to allow the formation of cell colonies. HBL-resistant clones from different plates are from different origins, thus are independent clones. A set of independent colonies from different plates was isolated, followed by PCR amplification and Sanger DNA sequencing of DNA fragments containing sgRNAs using the primers listed in S1 Table.

LITAF/CDIP1-dKO HT1080 cells and CAV1/LITAF/CDIP1-tKO HT1080 cells were made by CRISPR gene editing using the sgRNAs listed in S1 Table.

### Cell culture, transfection, and cytotoxicity assays

MEFs, BMDMs, and HT1080 cells were cultured in DMEM supplemented with 10% fetal bovine serum (FBS) and antibiotics (penicillin and streptomycin). MEFs were isolated as described previously [57]. MEF-WT cells and MEF-dKO (*Litaf*^−/−^/*Cdip1*^−/−^) cells were immortalized (iMEFs) by infection with a recombinant lentivirus containing both large and small SV40 T antigens (Lenti-SV40 Tta virus) (Cat. CIP-0014, Capital Biosciences) as described [26]. BMDMs were isolated as described previously [58,59]. BMDMs were cultured in L929 conditioned DMEM with 10% FBS. IECs were isolated from within 6-day newborn mice using IntestiCult OMG Mouse kit (#6005, StemCell Technologies) following the manufacturer’s manual.

For cytotoxicity assays, cells grown in 96-well plates with a 50%–80% confluence were incubated with various concentrations of HBL (1,000 ng/mL HBL defined as 1,000 ng/mL each of HBL-B, HBL-L1, and HBL-L2) for lengths of time as indicated. Cell viabilities were then assessed by MTT (3-[4,5-dimethylthiazol-2-yl]-2,5-diphenyltetrazolium bromide, Sigma Cat. No. M5655) assays as described previously [29], expressed as % of signals of untreated cells.

Full-length LITAF, CDIP1 and CAV1 cDNA from humans was amplified by PCR and cloned into the pIREShyg-2 mammalian expression vector [29]. DNA fragment containing CAV1-3C (CAV1 palmitoylation-deficient variant) was synthesized by GenScript. The primers used for cloning are listed in S1 Table. X-tremeGENE 9 DNA Transfection Reagent (Roche, Cat. No.: 06366236001) was used for transfection of the plasmids into the indicated cells following the manufacturer’s manual.

### Cell binding and Western blotting

For assessing HBL binding to cells, cells were grown to 80% confluence in 12-well plates and were incubated with HBL toxin or HBL-B for various lengths of time. Cell lysates were prepared in the modified Radioimmunoprecipitation assay (RIPA) lysis buffer containing protease inhibitors (EDTA-free Protease Inhibitor Cocktail) (# 4693132001, Sigma), and separated on SDS-PAGE and analyzed by Western blotting using antibodies as indicated.

In some experiments for assessing detergent-resistant membrane fractions (DRMs), cells were lysed on ice using Triton X-100 lysis buffer (50 mM Tris-HCl, pH 7.5, 150 mM NaCl, 1 mM EDTA, 1% Triton X-100) supplemented with protease inhibitors (# 4693132001, Sigma). Cell lysates were spun at 12,000 rpm for 10 min to separate Triton X-100 solubilized fractions (supernatants, S) and the detergent-resistant membrane fractions (DRMs) (pellets, P). The P and S fractions were boiled in 1 x SDS loading buffer, analyzed by Western blotting using antibodies as indicated.

In some experiments assessing the effects of removal of GPI-anchored proteins from cell surface on HBL-B binding to the cells, the cells were incubated with or without 1 µg/mL of HBL-B for 4 h at room temperature (23°C). Cells were then washed and incubated with PI-PLC (#P5542, Sigma) (0, 0.2, and 1.0 unit/mL) for 1 h at room temperature. After washing with PBS, cells were then lysed on ice using Triton X-100 lysis buffer. Cell lysates were spun at 12,000 rpm for 10 min to separate Triton X-100 solubilized fraction (supernatant, S) and the detergent-resistant membrane fractions (pellets, P). The P and S fractions were boiled in 1 x SDS loading buffer, analyzed by Western blotting using antibodies as indicated.

For immunoblotting analyses, cell lysates were separated on SDS-PAGE gels, transferred onto nitrocellulose membranes, and incubated with a primary antibody at 4°C overnight with rocking: rat anti-HBL-B, rat anti-HBL-L1, or rat anti-HBL-L2 antiserum (all in 1:5000 dilutions, made in our laboratory), anti-CAV1 (Cat#Ab2910, Abcam) (1:1000), anti-LITAF (# AF4695, R&D Systems) (1:500), anti-CDIP1 (# 13824S, Cell Signaling Technologies) (1:500), anti-MEK2 (#67410, Proteintech) (1:10000), anti-CD59 (#10742–1-AP, Proteintech) (1:1000), anti-tubulin (#66031, Proteintech) (1:20000) antibody, anti-β-actin (#660008, Proteintech) (1:5000). A donkey anti-mouse, rat, rabbit or other corresponding species secondary antibody conjugated with Alexa Fluor 488- or Alexa Fluor 680 fluorescent (1:5000–1:10000) (Thermo Fisher) was used for 2 h at room temperature to detect the targeted protein band. Fluorescent protein bands were imaged by using ChemiDoc MP Imaging System (Bio-Rad).

### Confocal microscopy

Cells grown on coverslips with 50% confluence were incubated with HBL-B for 2 h at 37°C. Cells were then washed three times with PBS, fixed in PBS containing 4% paraformaldehyde (wt/vol) at room temperature for 10 min. After washing with PBS three times, cells were permeabilized in 0.1% Triton X-100 (vol/vol) at room temperature for 30 min. After washing with PBS, cells were incubated with blocking buffer (containing 10% FBS, 1% BSA in PBS) at room temperature for 60 min. Cells were then incubated with primary antibodies in blocking buffer at 4°C overnight.

Rabbit polyclonal anti-CAV1 (Cat#Ab2910, Abcam) (1:500 dilution) and rat anti-HBL-B antiserum (1:1000 dilution) were used as primary antibodies. After washing with PBST (PBS with 0.1% Tween-20) three times for 5 min each, cell coverslips were incubated in PBST containing Alexa Fluor 488 donkey anti-rat (#A21208, Thermo Fisher) or Alexa Fluor 680 donkey anti-rabbit (#A32802, Thermo Fisher) (1:1000) at room temperature for 60 min. After washing with PBST three times, cell coverslips were mounted using Mowiol-DAPI (4′, 6-diamin-2-phenylindole-dihydrochloride) mounting medium for 1 h. Confocal microscopy analysis was carried out with a Leica SP8 LIGHTNING confocal system using the built-in software Leica Application Suite X 3.5.5.19976. All images in the same figure panel were taken under the same software setting. Separate images of stained cells were acquired for the green (HBL-B) and red (CAV1) fluorescence channels. These images were then merged to visualize colocalization, indicated by yellow regions in the composite images. To quantify this colocalization, intensity profiles for each channel were generated along selected regions of interest using ImageJ’s RGB Plot Profile tool. Background fluorescence was measured in cell-free areas of the same image and subtracted from cellular measurements to yield background-corrected fluorescence values. Overlapping peaks in the intensity curves from both channels suggest spatial coexistence of the two proteins.

### co-IP and protein interaction

For HBL and CAV1 co-immunoprecipitation experiments, iMEFs grown in 15-cm dishes were incubated with HBL-B (1,000 ng/mL) for 4 h at 23°C. After washing with PBS, cells were incubated with or without the protein crosslinker DTSSP (3,3′-dithiobis (sulfosuccinimidyl propionate), Sigma Cat. No. 803200) (1.2 mM) in PBS at 4°C for 30 min. DTSSP is a water-soluble and plasma membrane-impermeable crosslinker that crosslinks surface proteins that physically associate. It contains amine-reactive NHS-ester ends (12.0 Å in distance), and its central disulfide bond can be cleaved with reducing agents (such as DTT-containing SDS-PAGE sample loading buffer), allowing separation of crosslinked proteins. Cells were then washed, and cell lysates prepared in Triton X-100 lysis supplemented with EDTA-free Protease Inhibitor Cocktail (Sigma Cat. No. 4693132001) at room temperature. CAV1 is a detergent-resistant membrane-associated protein and can be extracted at room temperature rather than at lower temperature (0 and 4°C). Immunoprecipitation was performed using SureBeads Protein G Magnetic Beads (#1614023, BIO-RAD) bound with either a rabbit anti-CAV1 antibody (Abcam, Cat. No. AB2910) or a rat anti-HBL-B antiserum. The immunoprecipitated proteins were then analyzed by Western blotting using the anti-CAV1 antibody and anti-HBL-B antiserum.

For co-immunoprecipitation of HBL-B and LITAF or HBL-B and CDIP1, cells were incubated with HBL-B (1,000 ng/mL) at 4°C for 2 h. Cell lysates were then prepared as above, and immunoprecipitated using SureBeads Protein G Magnetic Beads (BIO-RAD, Cat. No. 1614023) bound with a goat anti-LITAF antibody (R&D, Cat. No. AF4695) or a rabbit anti-CDIP1 antibody (Cell Signaling Technologies, Cat. No. 13824S). The immunoprecipitated proteins were then analyzed by Western blotting using our rat anti-HBL-B antiserum, goat anti-LITAF, or rabbit anti-CDIP1 antibody. Anti-HBL-B, -L1, L2 antisera were prepared by immunization of rats via subcutaneous injections of HBL-B, -L1, L2, respectively.

To assess potential direct interactions, HBL-B or its β‑tongue deletion variant and CAV1 (Proteintech Cat. Ag0849), either individually or in combination (0.1 μg each), were incubated in 0.1 mL PBS or 0.1% DDM (n-Dodecyl-β-D-maltoside; D5172-1G, Sigma) at room temperature for 30 min. The mixtures (20 μl) were then resolved on a native gel, transferred to nitrocellulose membranes, and analyzed by Western blotting using anti-CAV1 (Cat#Ab2910, Abcam; 1:1000) and rat anti-HBL-B (1:5000 dilution) sequentially. A donkey anti-rat secondary antibody conjugated with Alexa Fluor 488 or a donkey anti-rabbit secondary antibody conjugated with Alexa Fluor 680 (1:5000–1:10000; Thermo Fisher) was used to detect HBL-B and CAV1, respectively. Fluorescent protein bands were imaged using the ChemiDoc MP Imaging System (Bio-Rad).

### Mouse studies

Mice are housed in cages with 24-h free access to food and water, with each cage holding a maximum of 4 male or 5 female mice at constant temperature (23°C) and humidity (55% ± 10%) with a 12-h light/dark cycle (lights on 7:00 am). Mice with indicated phenotypes in C57BL/6 background, 8–12-week-old, both male and female, were used. To avoid the potential degradation of the protein toxin by gastric acid and enzymes, we chose I.V. or I.P. route rather than oral gavage for HBL toxin administration to recapitulate the toxin pathogenic potential. Thus, these toxin challenge models are designed to mimic stages of infection in which *B. cereus* has already breached epithelial barriers or disseminated systemically, conditions under which HBL becomes biologically relevant. Therefore, in HBL challenge experiments, 8–12-week-old male and female mice with various genotypes were injected with one to three doses of HBL toxin. The mice were grouped based on genotypes. When mice of the same genotype received different treatments, they were randomly assigned to treatment groups. All toxin-challenged or infected mice were monitored twice daily for 1-week post-challenge for signs of malaise or mortality. I.P. route is a convenient way for multiple systemic administrations in mice for consecutive days. The toxin injected into peritoneal cavity can be taken up rapidly (< 30 min) into circulation via portal vein. Although toxin can reach circulation immediately via I.V. route, it is technically difficult to do multiple injections since mouse tail veins are thin and fragile and are easily damaged by first injection, making the following injections extremely hard. Therefore, we use I.P. route when repeated dosing is necessary.

### Statistical analysis

Statistical significances of differences were calculated using the two-tailed Student *t* test. Survival curves were compared via Log-rank Mantel-Cox tests. When more than two groups were compared, pairwise log-rank tests were performed following an overall group comparison. *P* values from pairwise comparisons were adjusted using the Holm procedure to control the family-wise error rate (using the built-in functions of the R (version 4.6.0): https://www.R-project.org/), and the adjusted *P* values were used to determine statistical significance. *P < 0.05* was considered as a significant difference. Other data represent mean values ± SD of at least three independent biological replicates.

## Supporting information

S1 Fig Susceptibility of CDIP1-KO mice to HBL toxin challenge. WT mice and *Cdip1*-null mice were challenged with 1 µg HBL toxin via intraperitoneal injection. Then survival was monitored. Log-rank test. The numerical data underlying this figure can be found in S1 Data.
(EPS)

S2 Fig HBL-B component is essential for CAV1-mediated HBL toxin’s cytolytic activity. **(A)** HBL-B component is essential for CAV1-mediated HBL toxin’s cytolytic activity. iMEF-tKO (+CAV1) and iMEF-dKO cells, HT1080-WT and HT1080-dKO cells were incubated with 0–1,000 ng/mL HBL (0–1,000 ng/mL each of HBL-B, HBL-L1, and HBL-L2), or 0–1,000 ng/mL of HBL-L1 plus HBL-L2 (without HBL-B) for 4 h, followed by an MTT assay to assess cell viability. Of note, HBL-B component is absolutely required for the toxin’s cytolytic activity to all these cells. Mean ± SD of three independent biological replicates. **(B)** No cytolytic activity of single HBL components. HT1080-WT and iMEF-WT cells were incubated with various concentrations of HBL or each individual HBL components for 24 h, followed by an MTT assay to assess cell viability. Of note, HBL components are individually no cytolytic activity to these cells. Mean ± SD of three independent biological replicates. The numerical data underlying this figure can be found in S1 Data.
(EPS)

S3 Fig HBL cytolytic activity is independent of endocytic processes. **(A)** iMEF-WT, iMEF-dKO, HT1080-WT, and HT1080-dKO cells were pre-incubated with/without endocytosis inhibitors ammonium chloride (NH_4_Cl) (10 mM) or bafilomycin A1 (Baf-A1) (0.1 µM) for 20 min. Then the cells were further incubated with various concentrations of HBL toxin for 4 h, followed by an MTT assay to measure cell viability. Mean ± SD of three independent biological replicates. **(B)** The cells were pretreated with/without endocytosis inhibitors as in (A). Then the cells were further incubated with modified anthrax toxin (0–1,000 ng/mL PA + 100 ng/mL FP59) for 24 h, followed by an MTT assay to evaluate cell viability. Mean ± SD of three independent biological replicates. Of note, while NH_4_Cl and bafilomycin A1 could inhibit the cytotoxicity of PA + FP59, these inhibitors could not alter the HBL-mediated cytolytic activity to the cells. **(C**, **D)** iMEFs and HT1080 cells were cooled down at 4°C prior to added with various concentrations of HBL toxin (**C**) or modified anthrax toxin (PA + FP59) (**D**). Cells were then moved to 15°C or 37°C, were further incubated for 24 h, followed by an MTT assay to evaluate cell viability. Mean ± SD of three independent biological replicates. Of note, the cytotoxicity of PA + FP59 was completely inhibited at 15°C (the endocytosis-impermissive low temperature), whereas the cytolytic activity of HBL toxin remained largely unaffected. The numerical data underlying this figure can be found in S1 Data.
(EPS)

S4 Fig Removal of GPI-anchored proteins from cell surface does not affect HBL-B binding to the cells. HT1080-WT and HT1080-dKO cells were incubated with or without 1 µg/mL of HBL-B for 4 h at room temperature (23°C). Cells were then washed and incubated with PI-PLC (0, 0.2, and 1.0 unit/mL) for 1 h at room temperature. After washing, cells were then lysed on ice using Triton X-100 lysis buffer. Cell lysates were spun at 12,000 rpm for 10 min to separate Triton X-100 solubilized fraction (supernatant, S) and the detergent-resistant membrane fractions (pellets, P). The P and S fractions were boiled in 1 x SDS loading buffer, analyzed by Western blotting using antibodies as indicated. Of note, PI-PLC could greatly reduce association of CD59 with cells. Cytosol protein MEK2 was mostly located in Triton X-100 solubilized fraction. Apparently, the association of HBL-B and CAV1 could not withstand the Triton X-100 extraction and thus HBL-B was mostly found in Triton X-100 solubilized fraction.
(EPS)

S5 Fig CAV1 does not affect the function of other HBL receptors. Primary ECs and BMDMs isolated from WT and *CAV1*^*−/−*^ mice were incubated with various concentrations of HBL toxin for 4 h, followed by an MTT assay to evaluate cell viability. Of note, all the *CAV1*-KO cells remain similar sensitivity as their WT cells to HBL toxin. Mean ± SD of three independent biological replicates. The numerical data underlying this figure can be found in S1 Data.
(EPS)

S6 Fig Co-localization of HBL-B and CAV1 on the cell surface. **(A, B)** HT1080-WT (A) and HT1080-dKO (B) cells bound with HBL-B were stained with an anti-HBL-B antiserum (green) and an anti-CAV1 antibody (red) as in Fig 3 and the images were merged to visualize colocalization (yellow) (only composite images shown). To quantify colocalization, intensity profiles for each channel were generated along selected regions of interest (white arrows) using ImageJ’s RGB Plot Profile tool (scanned along the arrows). Overlapping peaks in the intensity curves from both channels suggest spatial coexistence of the two proteins on cell surface. Notably, colocalizations were predominantly observed at the cell surface. Scale bar = 25 μm. The numerical data underlying this figure can be found in S1 Data.
(EPS)

S7 Fig Co-localization of HBL-B and CAV1 on the cell surface. Co-localization (yellow) of HBL-B (green) and CAV1 (red) on iMEF cell surface. iMEF-WT and iMEF-tKO cells bound with HBL-B were stained with an anti-HBL-B antiserum (green) and an anti-CAV1 antibody (red). Merge images highlight co-localization (yellow) of HBL-B and CAV1. DAPI-stained nuclei (blue). Scale bar = 25 μm.
(EPS)

S8 Fig Expression and secretion of the HBL-B β-tongue variants by the BH500 host strainstrain. HBL-B β-tongue variants, including HBL‑B (I245A), HBL‑B (G246A), HBL‑B (G247A), HBL‑B (I248A), and a β‑tongue deletion mutant HBL‑B Δ(I239–V252), were generated using In-Fusion cloning. These variants were expressed in the BH500 host strain using pYS5‑based expression vectors, and the recombinant proteins were secreted into the culture medium in their active form. Culture supernatants (10 µl or 5 µl) from each variant were analyzed by SDS-PAGE and stained with Coomassie blue. Notably, all variants were efficiently expressed and secreted at levels comparable to WT HBL‑B, without detectable degradation, indicating that these β‑tongue mutations do not compromise protein stability.
(EPS)

S9 Fig The palmitoylation-deficient CAV1 variant (CAV1-3C) is unable to mediate cytolytic activity of HBL toxin. (**A**) iMEF‑tKO cells reconstituted with either CAV1‑WT or the palmitoylation‑deficient CAV1 variant (CAV1‑3C) were lysed on ice using Triton X‑100 lysis buffer. Cell lysates were centrifuged to separate Triton X‑100-soluble fractions (supernatants, S) from detergent‑resistant membrane (DRM) fractions (pellets, P). The P and S fractions were boiled in 1× SDS loading buffer and analyzed by Western blotting using the indicated antibodies. The GPI‑anchored protein CD59 was exclusively detected in DRMs, while tubulin was predominantly found in Triton X‑100-soluble fractions. (B) Quantification of the pellet/supernatant ratio for CAV1‑WT versus CAV1‑3C from (A). The ratios of CAV1 protein band intensities in pellet and supernatant from (A) were estimated using ImageJ. As expected, most CAV1‑WT localized to DRMs, whereas CAV1‑3C showed a markedly reduced ability to partition into DRMs. (C) iMEF‑tKO (+ CAV1‑3C) and iMEF‑tKO (+ CAV1‑WT) cells were treated with various concentrations of HBL for 4 h, followed by an MTT assay to assess cell viability. While CAV1‑WT restored HBL sensitivity in iMEF‑tKO cells, all iMEF‑tKO cells expressing CAV1‑3C remained resistant to the toxin. In CAV1‑3C #2 cells, CAV1 expression was undetectable. Data represent mean ± SD from three independent biological replicates. The numerical data underlying this figure can be found in S1 Data.
(EPS)

S10 Fig Co-immunoprecipitation of HBL-B with CAV1, LITAF, and CDIP1. **(A)** iMEF-WT cells were incubated with or without HBL-B (1 µg/mL) for 4 h at room temperature (23°C). After washing, cells were incubated with or without a protein crosslinker DTSSP (1.2 mM) in PBS for 1 h at 4°C. DTSSP is a water-soluble and plasma membrane-impermeable protein crosslinker (8-atom spacer arm, 12.0 Å), allowing for crosslinking of cell surface proteins that interact. Cell lysates prepared in Triton X-100 lysis buffer were immunoprecipitated using a rabbit anti-CAV1 antibody or a rat anti-HBL-B antiserum. Then the immunoprecipitated samples were analyzed by Western blotting using the rabbit anti-CAV1 antibody and the rat anti-HBL-B antiserum. Of note, the physical association of HBL-B and CAV1 could be detected even in the absence of crosslinker DTSSP. **(B)** Co-immunoprecipitation of HBL-B with CDIP1. HT1080-WT and HT1080-dKO cells were incubated with or without HBL-B (1 µg/mL) for 2 h at 4°C. Then the cells were treated with or without DTSSP as in (A). Cell lysates were then prepared in Triton X-100 lysis buffer at room temperature, and CDIP1 was immunoprecipitated using a rabbit anti-CDIP1 antibody, followed by immunoblotting with a rat anti-HBL-B antiserum and rabbit anti-CDIP1 antibody. Of note, the physical association of HBL-B and CDIP1 did not occur in HT1080 (dKO) cells. **(C)** Co-immunoprecipitation of HBL-B with LITAF. iMEF-tKO (+LITAF) cells were incubated HBL-B (1 µg/mL) for 2 h at 4°C. Then the cells were treated with or without DTSSP as in (A). Cell lysates were then prepared in Triton X-100 lysis buffer at room temperature. LITAF was immunoprecipitated using a goat anti-LITAF antibody, followed by Western blotting analyses with a rat anti-HBL-B antiserum and a goat anti-LITAF antibody. Of note, the co-IP of HBL-B and LITAF could be detected in the absence of DTSSP.
(EPS)

S11 Fig Formation of CAV1 and HBL-B complex in solution. HBL‑B or its β‑tongue deletion variant, together with CAV1 (0.1 μg each), were incubated either individually or in combination in 0.1 mL PBS or 0.1% DDM at room temperature for 30 min. Mixtures (20 μL) were then resolved on a native gel, and potential complex formation was assessed by Western blotting using rabbit anti‑CAV1 and rat anti‑HBL‑B antisera. In PBS, CAV1 predominantly formed large aggregates that failed to migrate and remained in the loading wells. In contrast, CAV1 incubated in DDM migrated into the gel as smaller oligomeric species. HBL‑B appeared as high‑molecular‑weight oligomers under both PBS and DDM conditions. Because migration in native gels is influenced not only by molecular size but also by protein conformation and net surface charge, the protein ladders shown serve only to illustrate the gel’s separation efficiency. Notably, HBL‑B and CAV1 co‑migrated in DDM, forming a distinct protein complex. As expected, the HBL‑B β‑tongue deletion variant, which migrated substantially faster than WT HBL‑B, did not form a complex with CAV1.
(EPS)

S1 Table Primers used for PCR genotyping, cloning, and CRISPR editing. (DOCX)

S1 Data The numerical data underlying Figs 1C–1E, 2D–2G, 3B, 3C, 4B, 6 and S1, S2, S3, S5, S6, and S9. (XLSX)

S1 Raw Images Uncropped, unprocessed images for Figs 2C, 4C, 5, S4, S9A, S10, and S11. (PDF)

## Abbreviations

α-PFT: α-pore-forming toxin
CAV1: Caveolin-1
CDCs: cholesterol-dependent cytolysins
co-IP: co-immunoprecipitation
DRMs: detergent-resistant membrane fractions
ECs: endothelial cells
FBS: fetal bovine serum
GeCKO: genome-wide CRISPR/Cas9 knockout
GPI: glycosylphosphatidylinositol
iMEFs: immortalized MEFs
LITAF: LPS-induced TNF-alpha factor
MEFs: mouse embryonic fibroblasts
MOI: multiplicity of infection
NH_4_Cl: ammonium chloride
PA: protective antigen
PFTs: pore-forming toxins
WT: wildtype.
