# Supplementary material for: Caveolin 1 is a conserved host factor mediating Bacillus cereus hemolysin BL toxin cytolytic toxicity
Source: PLoS Biol. 2026 Aug 21;24(8):e3003957. doi: 10.1371/journal.pbio.3003957 (PMC13524335; doi:10.1371/journal.pbio.3003957)
Supplement: Supplementary file 1 [file pbio.3003957.s014.pdf]

Fig 2C

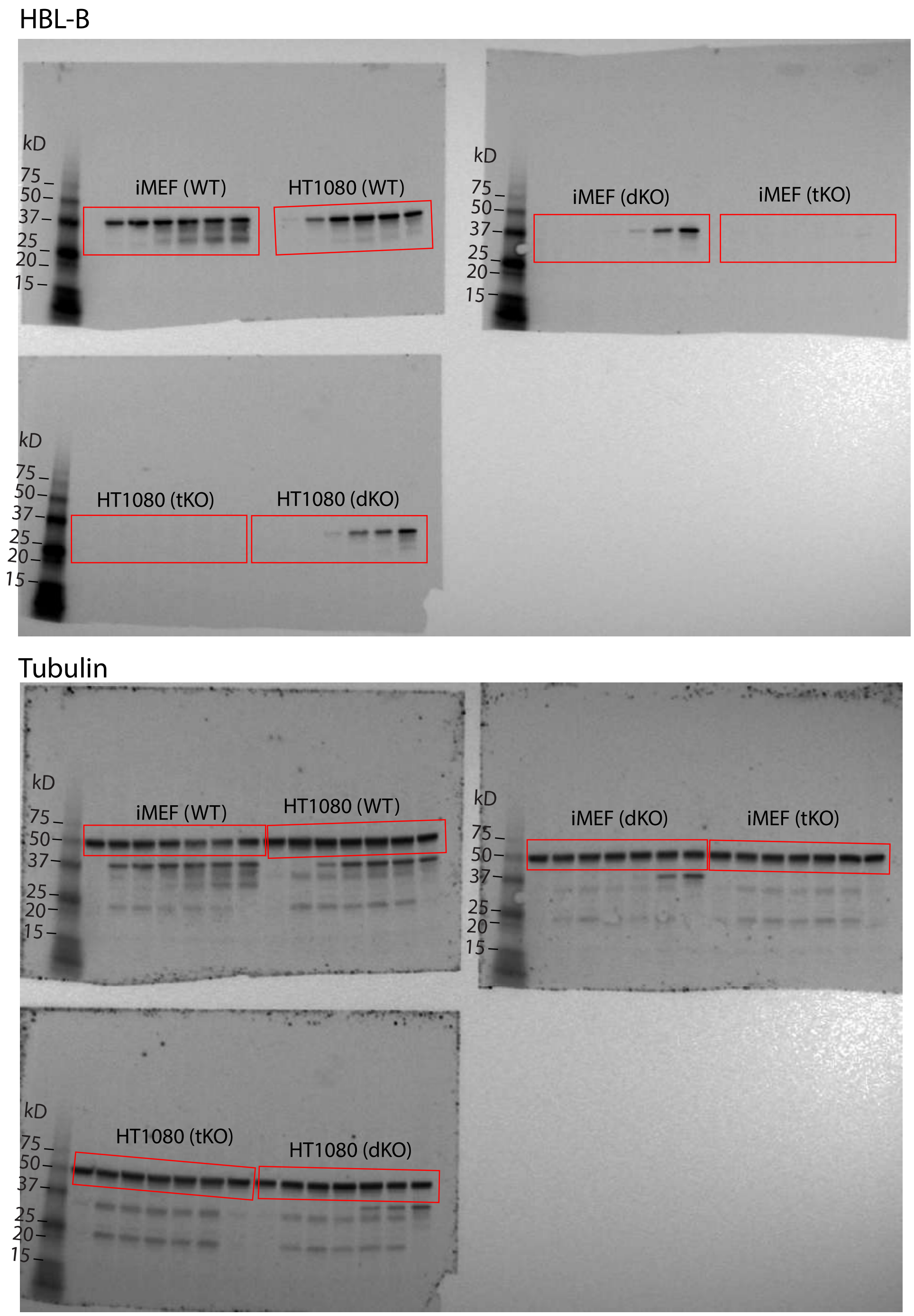
HBL-B
kD
75
50
37
25
20
15
iMEF (WT)
HT1080 (WT)
iMEF (dKO)
iMEF (tKO)
HT1080 (tKO)
HT1080 (dKO)
Tubulin
iMEF (WT)
HT1080 (WT)
iMEF (dKO)
iMEF (tKO)
HT1080 (tKO)
HT1080 (dKO)

Fig 4C

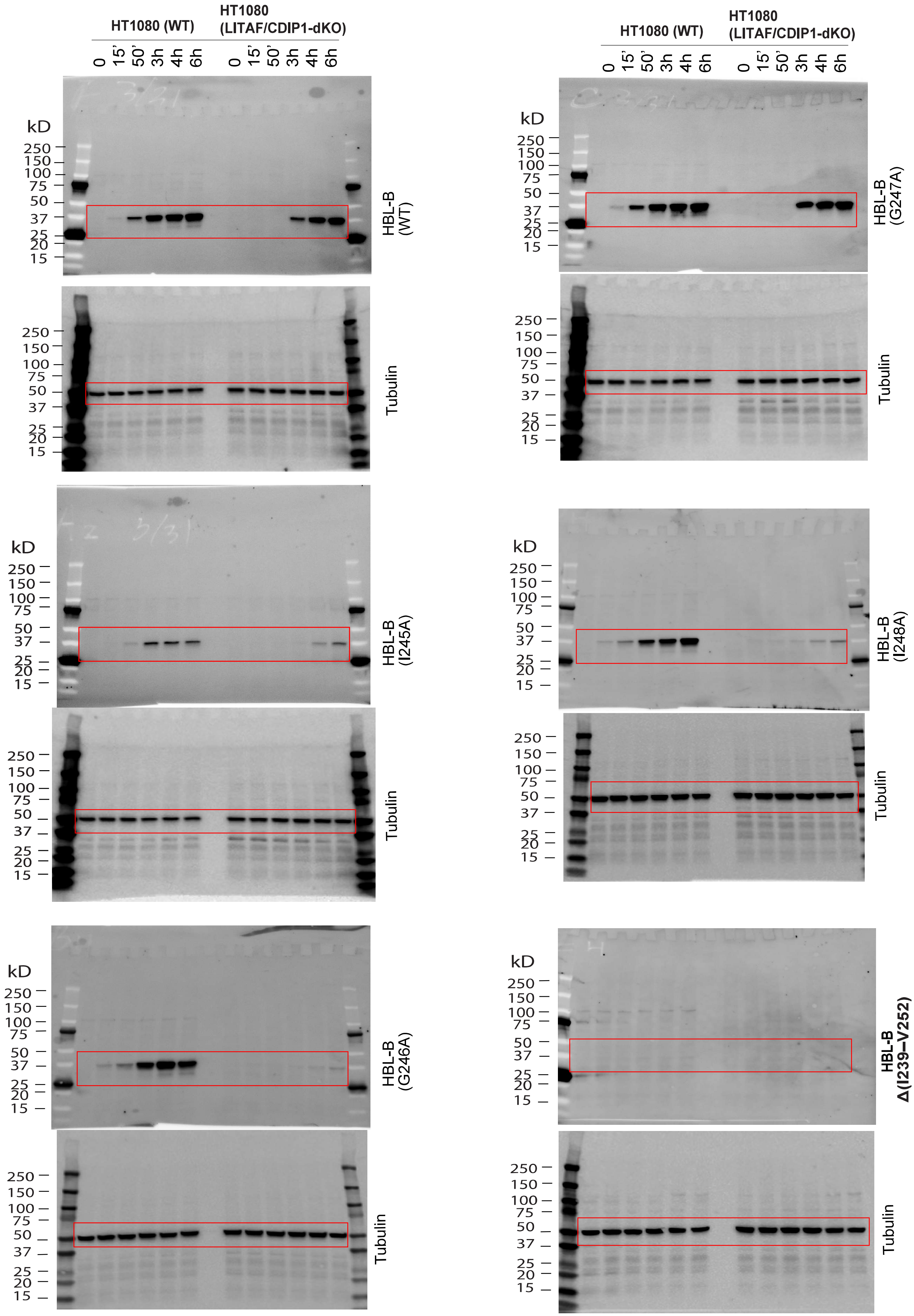

HT1080 (WT)
HT1080 (LITAF/CDIP1-dKO)
0
15'
50'
3h
4h
6h
0
15'
50'
3h
4h
6h
kD
250
150
100
75
50
37
25
20
15
HBL-B (WT)
Tubulin
HBL-B (G247A)
HBL-B (I245A)
HBL-B (I248A)
HBL-B (G246A)
HBL-B Δ(I239–V252)

Fig 5

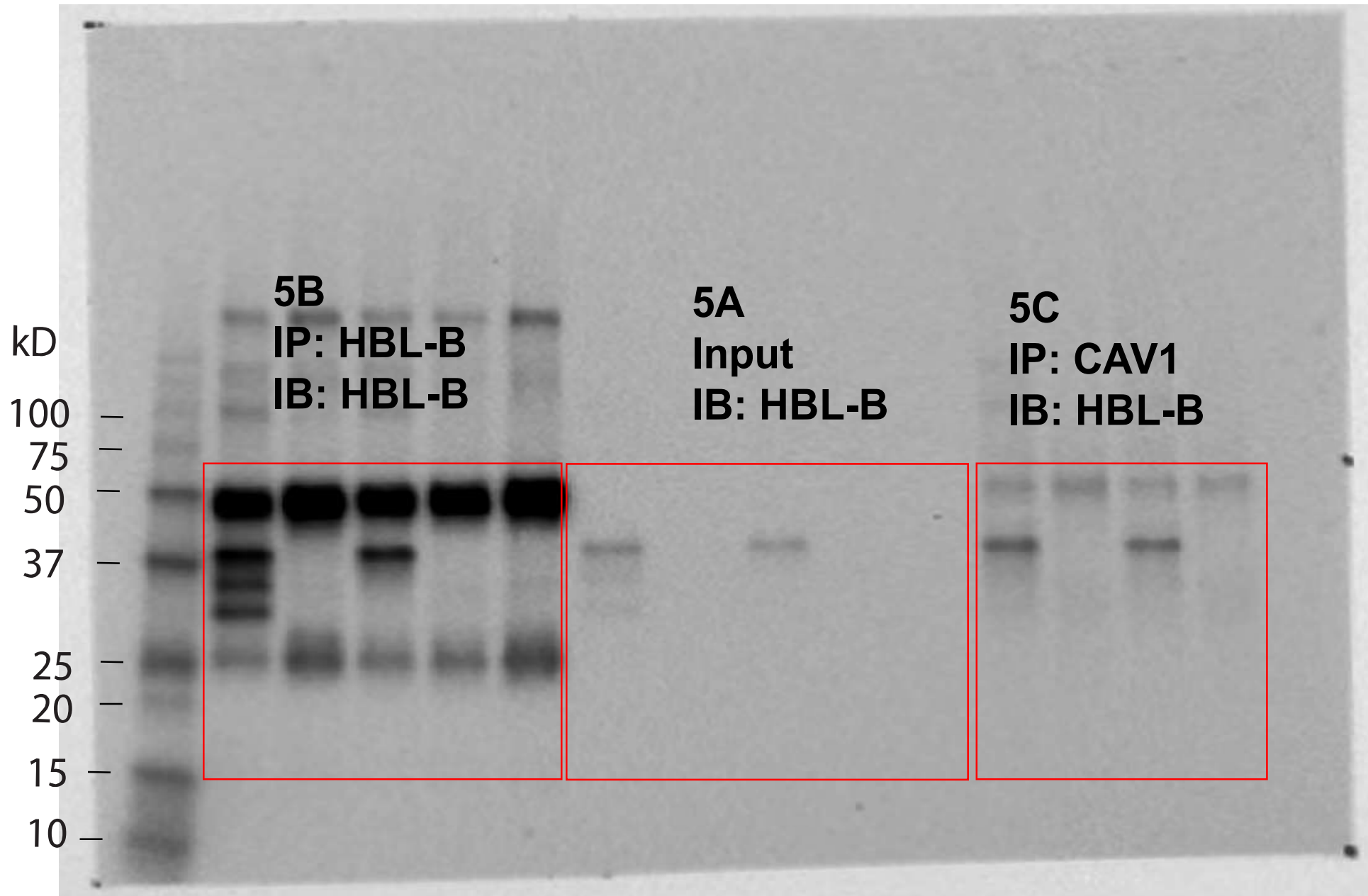
kD
100
75
50
37
25
20
15
10
5B
IP: HBL-B
IB: HBL-B
5A
Input
IB: HBL-B
5C
IP: CAV1
IB: HBL-B

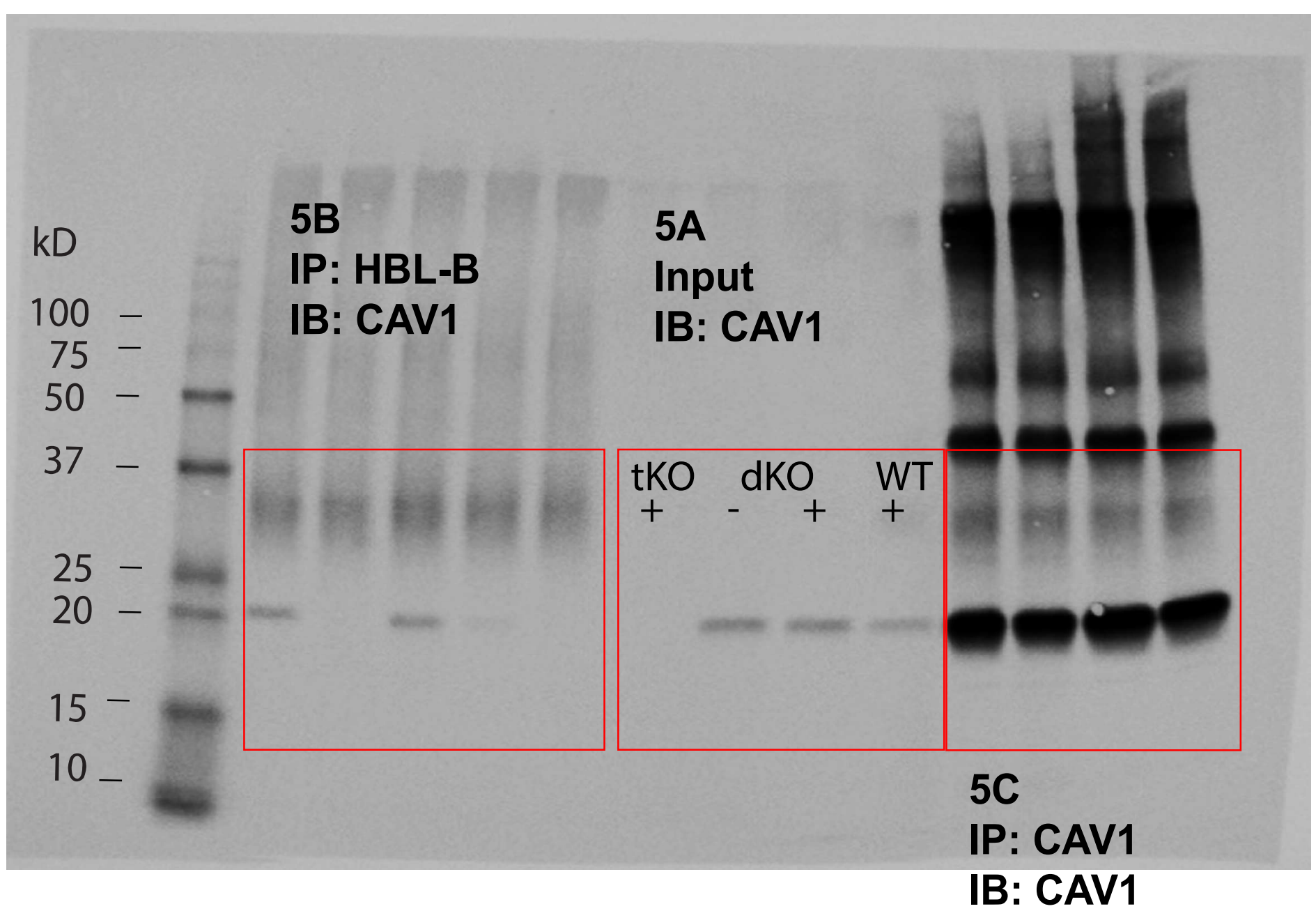
kD
100
75
50
37
25
20
15
10
5B
IP: HBL-B
IB: CAV1
5A
Input
IB: CAV1
tKO
dKO
WT
+
-
+
+
5C
IP: CAV1
IB: CAV1

S4 Fig

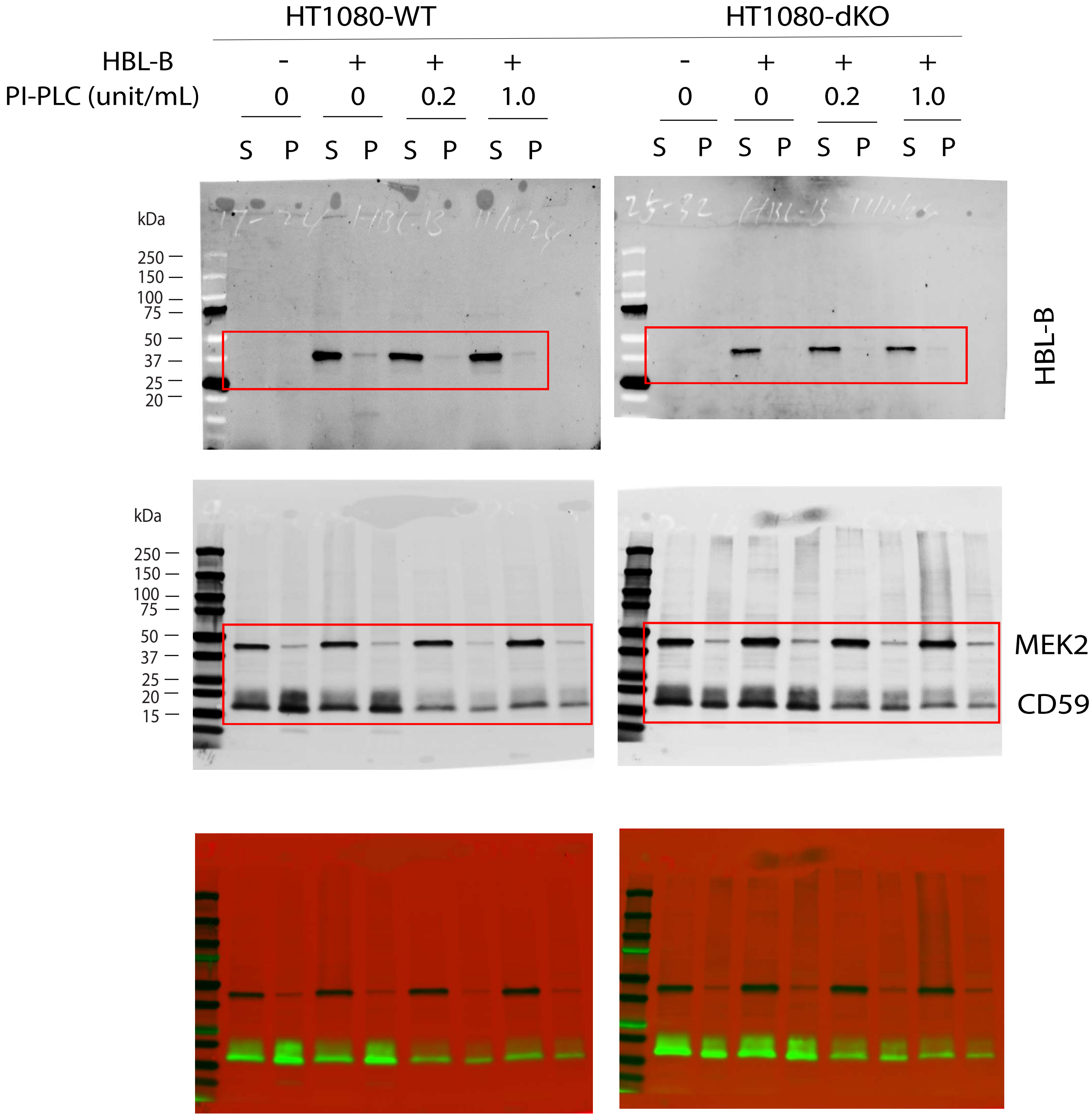
HT1080-WT
HT1080-dKO
HBL-B
PI-PLC (unit/mL)
- + + +
0 0 0.2 1.0
- + + +
0 0 0.2 1.0
S P S P S P S P
S P S P S P S P
kDa
250
150
100
75
50
37
25
20
15
HBL-B
MEK2
CD59

S8 Fig

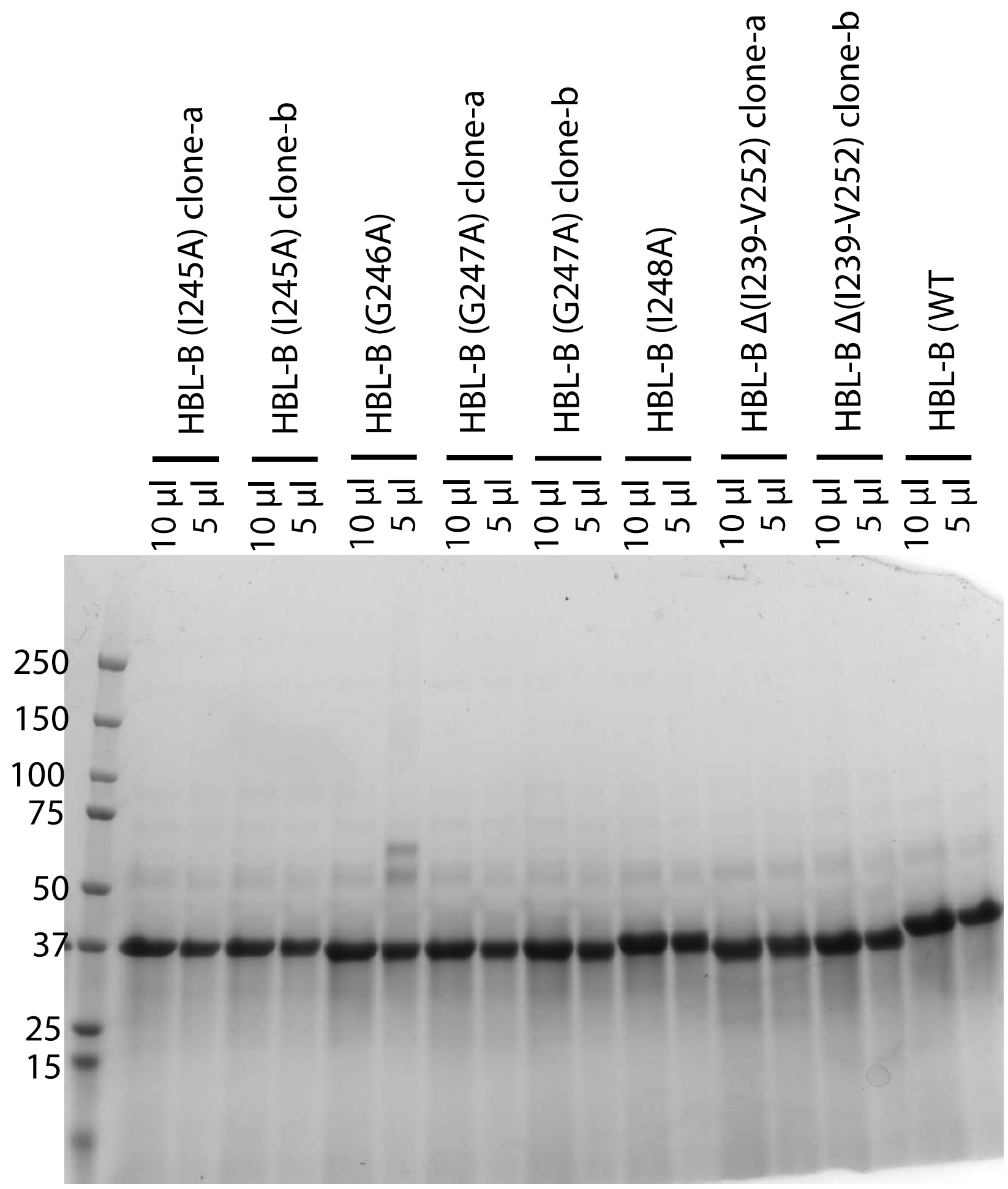
HBL-B (I245A) clone-a
HBL-B (I245A) clone-b
HBL-B (G246A)
HBL-B (G247A) clone-a
HBL-B (G247A) clone-b
HBL-B (I248A)
HBL-B Δ(I239-V252) clone-a
HBL-B Δ(I239-V252) clone-b
HBL-B (WT
10 µl
5 µl
250
150
100
75
50
37
25
15

S9A Fig

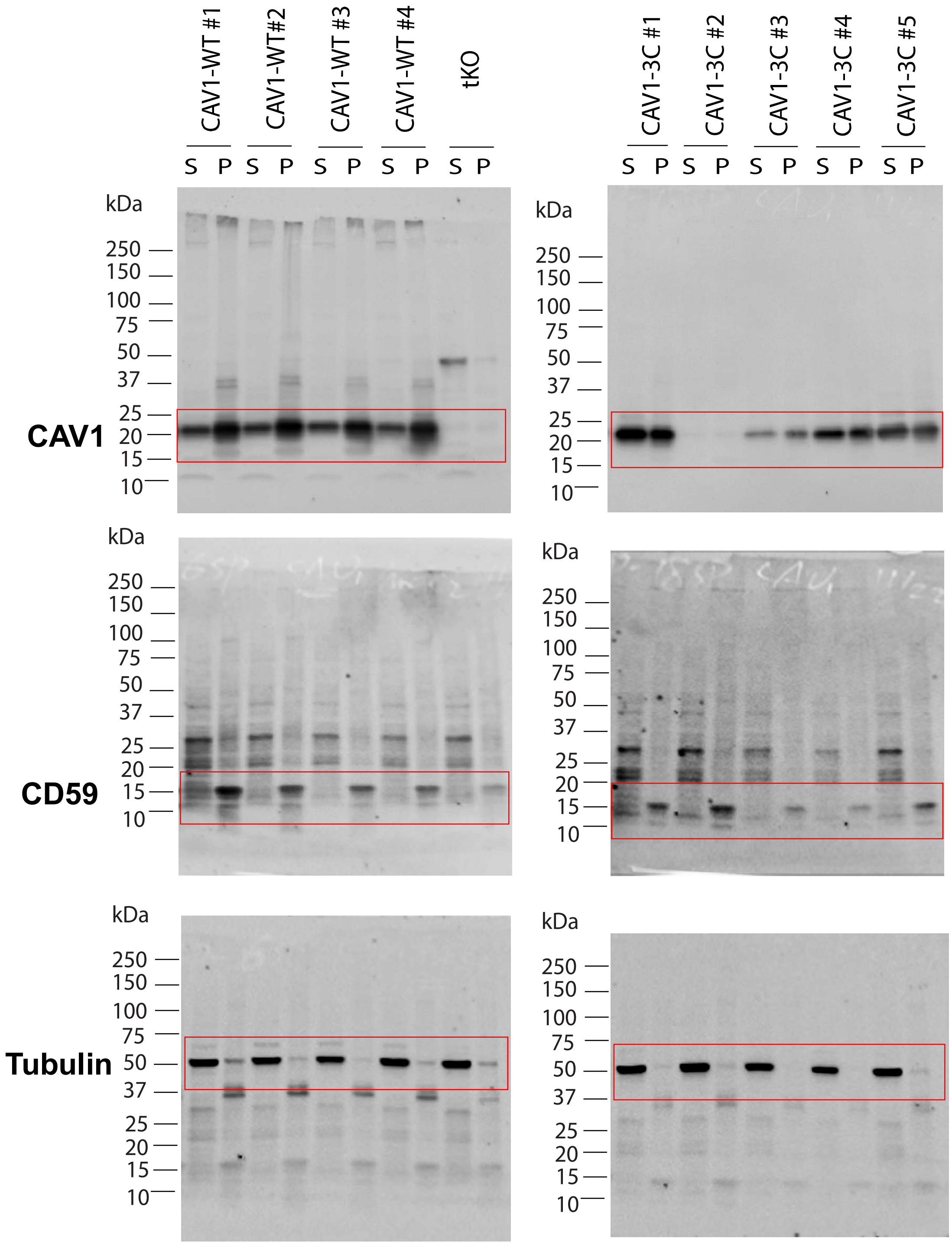
CAV1-WT #1
CAV1-WT#2
CAV1-WT #3
CAV1-WT #4
tKO
S P S P S P S P S P
CAV1-3C #1
CAV1-3C #2
CAV1-3C #3
CAV1-3C #4
CAV1-3C #5
S P S P S P S P S P
kDa
250
150
100
75
50
37
25
20
15
10
CAV1
CD59
Tubulin

S10 Fig

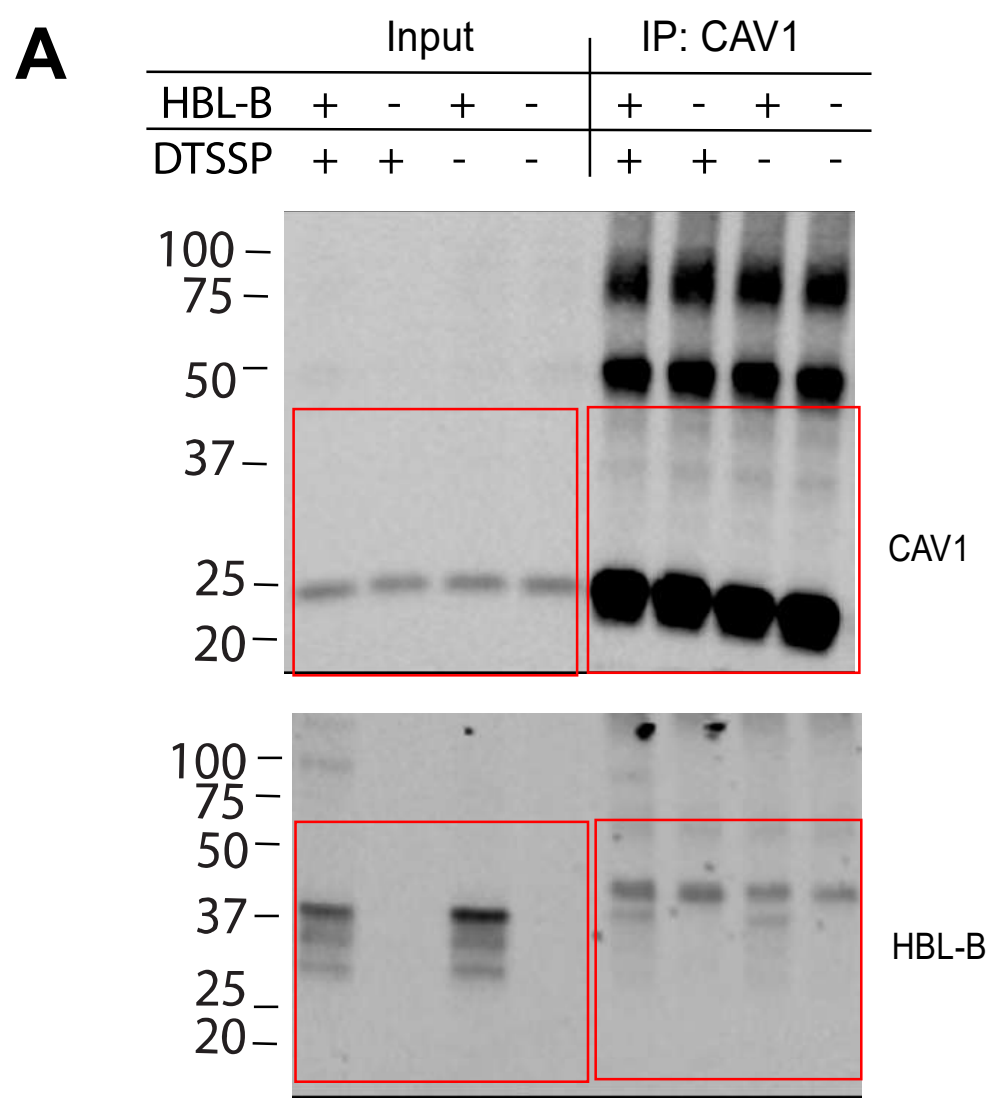

A
Input
IP: CAV1
HBL-B + - + - + - + -
DTSSP + + - - + + - -
100
75
50
37
25
20
CAV1
100
75
50
37
25
20
HBL-B

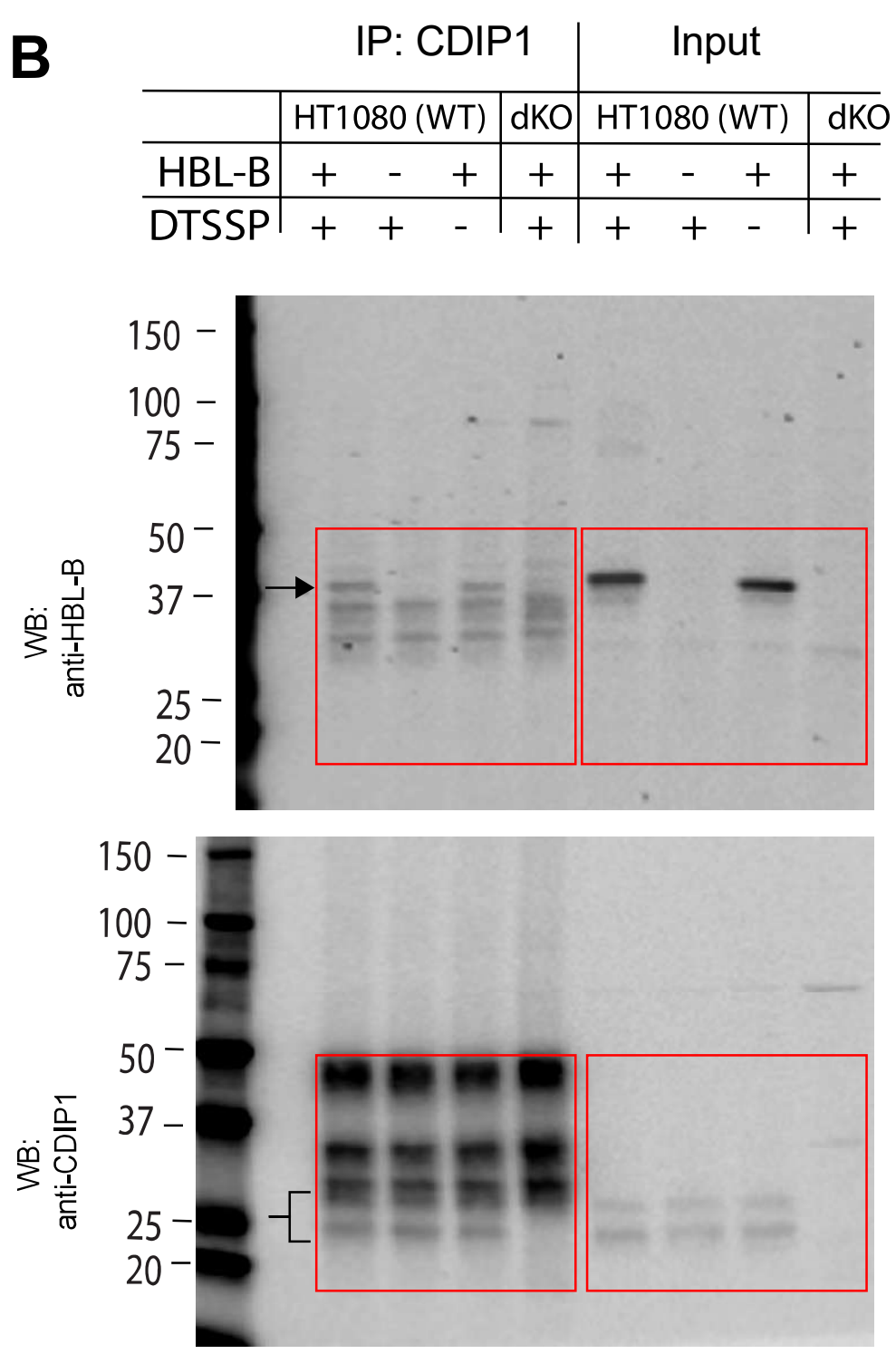

B
IP: CDIP1
Input
HT1080 (WT)
dKO
HT1080 (WT)
dKO
HBL-B + - + + + - + +
DTSSP + + - + + + - +
150
100
75
50
37
25
20
WB: anti-HBL-B
WB: anti-CDIP1

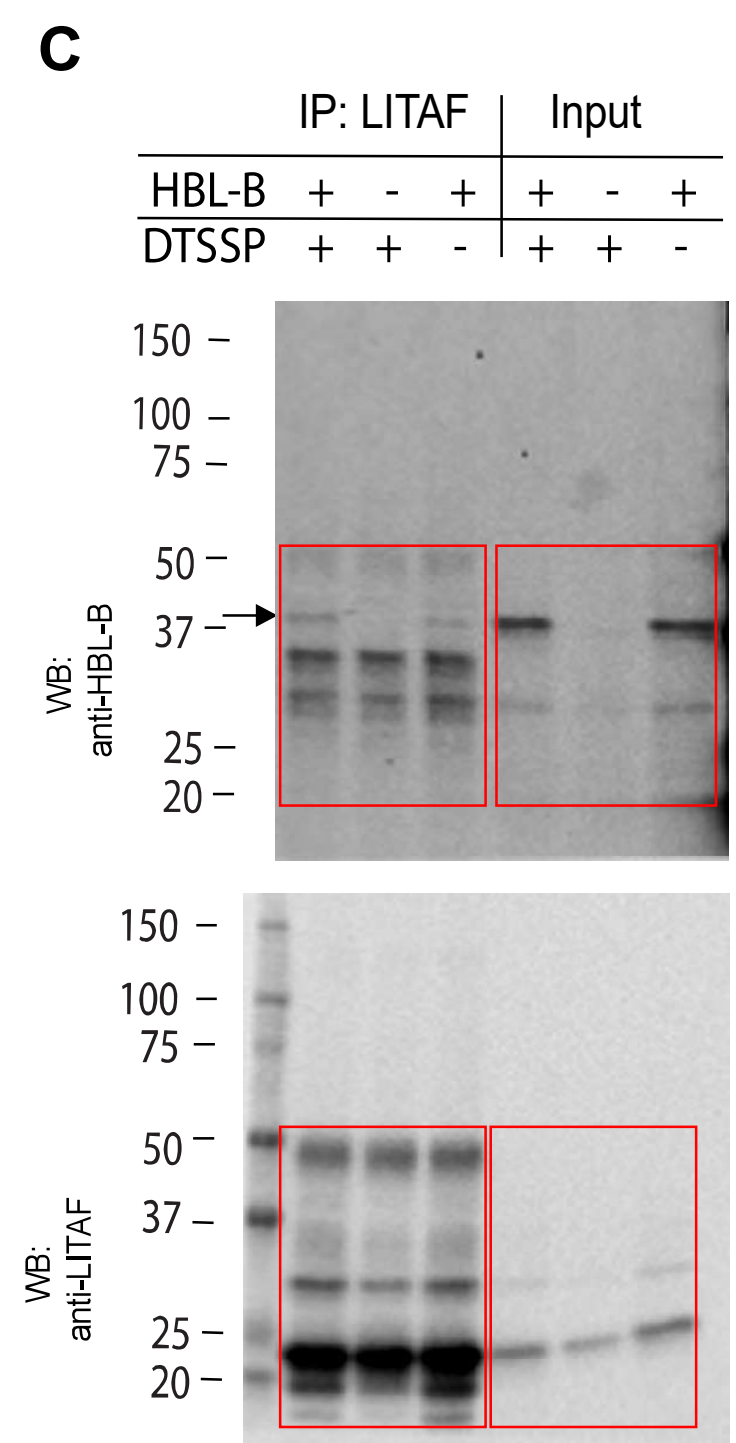

C
IP: LITAF
Input
HBL-B + - + + - +
DTSSP + + - + + -
150
100
75
50
37
25
20
WB: anti-HBL-B
WB: anti-LITAF

S11 Fig

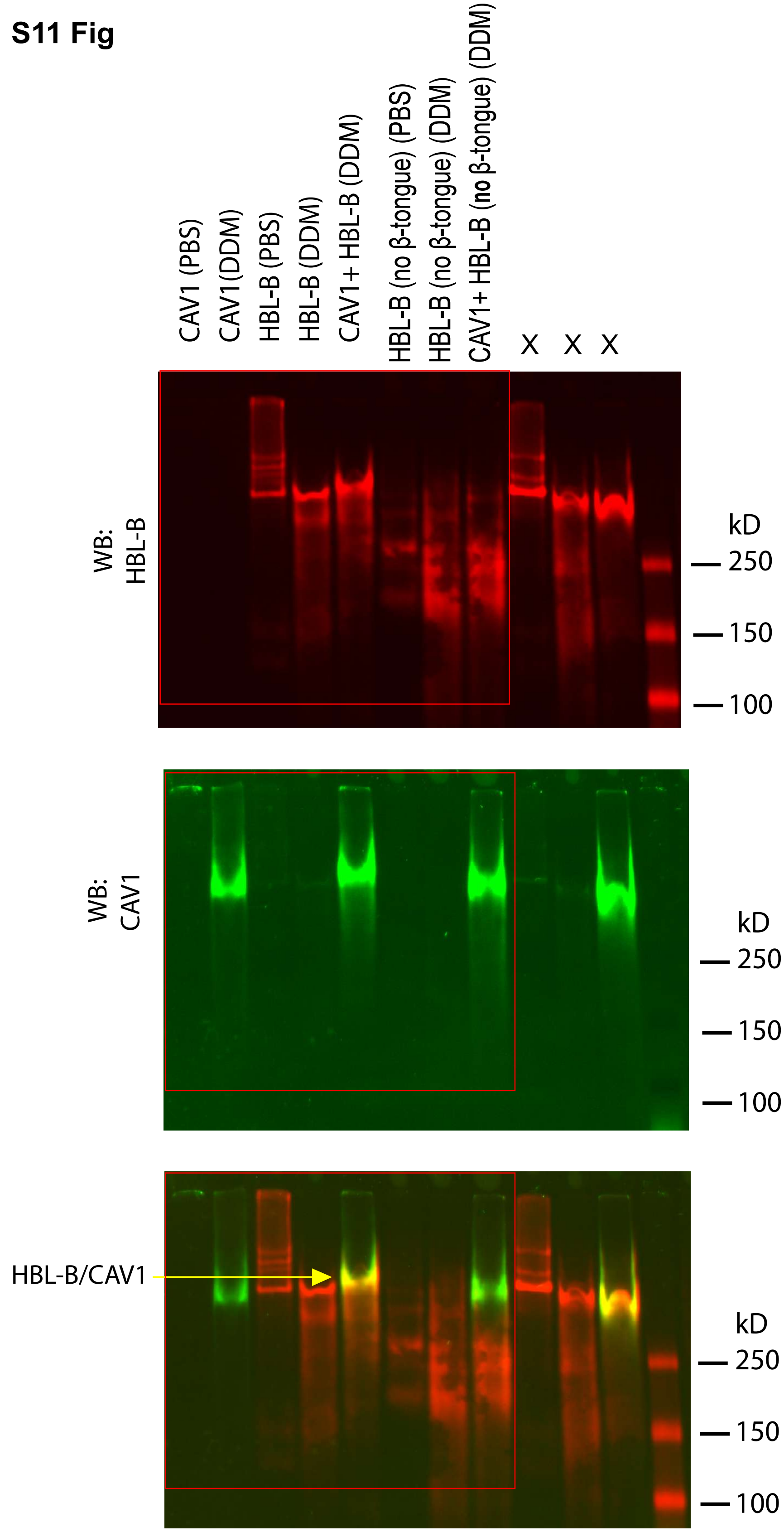

(Composite image)
