# Supplementary material for: Caveolin 1 is a conserved host factor mediating Bacillus cereus hemolysin BL toxin cytolytic toxicity
Source: PLoS Biol. 2026 Aug 21;24(8):e3003957. doi: 10.1371/journal.pbio.3003957 (PMC13524335; doi:10.1371/journal.pbio.3003957)
Supplement: Supplementary file 2 [file pbio.3003957.s012.docx]

**S1 Table.** Primers used for PCR genotyping, cloning, and CRISPR editing

| Use | Primer name and sequence | | Note |
| --- | --- | --- | --- |
| LITAF-allele genotyping | For | TGTCGGCTCCAGGACCTTACCAA | WT: For+Rev-1=334 bp  KO: For+Rev-2= 364 bp |
|  | Rev-1 | GGTGGACATCACAGTGTCTGTCA |  |
|  | Rev-2 | GCACAACTCCAGCAGCAGTTTCTG |  |
| CDIP1-allele genotyping | CDIP1-F1 | TATGGGTGACCATAGAGCAGCATAAAGGA | WT: CDIP1-F1+CDIP1-R1=209 bp  WT: CDIP1-F1+CDIP1-R2=1259 bp  KO: CDIP1-F1+CDIP1-R2=292 bp |
|  | CDIP1-R1 | AGCCTCAGGCTCAAACTCAGCTACCTGAGC |  |
|  | CDIP1-R2 | AGAGCTAGGAGTATATCGCTCTACTCCAT |  |
| CAV1-allele genotyping | WT-F | GCACACCAAGGAGATTGACC | WT-F+ Common-R=661 bp  Mut-F+ Common-R=200 bp |
|  | Mut-F | CTCCAGACTGCCTTGGGAAAA |  |
|  | Common-R | CTTGGCTGTCACCACACAC |  |
| PCR for sgRNA containing DNA fragments | For-1st | AATGGACTATCATATGCTTACCGTAACTTGAAAGTATTTCG | 347 bp (using For-1st for Sanger sequencing) |
|  | Rev-1st | CTTTAGTTTGTATGTCTGTTGCTATTATGTCTACTATTCTTTCC |  |
| Human CAV1 cDNA cloning/  expression | hCAV1-F | CGTAAAGAATTCGCCACCATGTCTGGGGGCAAATACGTAGACTCGG | EcoRI/BamHI sites of pIRESHgy-2  (Expression plasmid) |
|  | hCAV1-R | GGGGAGGTGGTCTGGATCCGCCGGCACCGGTTATATTTCTTTCTGCAAGTTGATGCGG |  |
| Human LITAF  CRISPR editing | For | CACCGAGTCAGCTGTCCTATAACGC | After annealing, cloned into BpiI-digested pSpCas9-based vectors |
|  | Rev | AAACGCGTTATAGGACAGCTGACTC |  |
| Human CDIP1  CRISPR editing | For | CACCGACCAAGATCTCCTACGAGAT | Same as above |
|  | Rev | AAACATCTCGTAGGAGATCTTGGTC |  |
| Human CAV1  CRISPR editing | For | CACCGAGGTCCTACAGCCCCACTAC | Same as above |
|  | Rev | AAAC GTAGTGGGGCTGTAGGACCT C |  |
| Mouse CAV1  CRISPR editing | For | CACCGCAAGCATCTCAACGACGACG | Same as above |
|  | Rev | AAACCGTCGTCGTTGAGATGCTTGC |  |
