# Supplementary material for: Caveolin 1 is a conserved host factor mediating Bacillus cereus hemolysin BL toxin cytolytic toxicity
Source: PLoS Biol. 2026 Aug 21;24(8):e3003957. doi: 10.1371/journal.pbio.3003957 (PMC13524335; doi:10.1371/journal.pbio.3003957)
Supplement: Supplementary file 4 [file pbio.3003957.s018.docx]

Point‑by‑point responses to the reviewers
----------------------------------------------------------------
**Reviewer #1** (Holger Barth):

In the present manuscript, the authors report about the identification and characterization of a third cellular receptor for the  tripartite α‑pore‑forming toxin and a major virulence factor hemolysin BL (HBL) from the medically relevant human pathogenic bacterium Bacillus cereus. Before, this consortium identified and characterized two other cellular membrane receptors for HBL, namely LITAF and CDIP1. They recognized that cells lacking these two receptors were still intoxicated by exposure to HBL, although not as efficient as wild-type cells. This prompted the authors to use double-knock out mouse and human cells to perform genome-wide CRISPR knockout screens to identify cells that are totally resistant towards HBL. The only hit found in these cells was caveolin 1 (CAV1), a trans-membrane protein. The authors then performed a elegant series of experiments including co-localization by fluorescence microscopy and co-precipitation / Western blot analysis to investigate
whether the binding subunit of HBL (HBL-B) interacts with CAV1 in cells, which was convincingly demonstrated by the results. All necessary controls were involved in these experiments and the conclusions drawn from these results are justified. Next, the interaction between HBL-B and Cav1 was analyzed in more detail. To this end, specific amino acid residues in the so-called ß-tongue region of HBL-B were modified and the resulting variations characterized and tested for binding to CAv-1 and for cytotoxicity of cells. Taken together, the importance of the ß-tongue for binding to CAV-1, which is mainly localized on the inner side of the plasma membrane was confirmed and residues I245, G246 and I 248 identified as crucial for the binding of HBL-B to Cav-1.  Again, all necessary controls were included and the results are convincing enough to support the conclusions. Amongst other detailed characterizations of the interaction of HBL with Cav1 and the other receptors, the authors
also excluded that CAV-1 interacts with the other two HBL receptors in cells. Finally, and importantly, the role of CAV-1 for HBL pathogenesis was confirmed in vivo by using a mouse model. Interestingly, the results obtained with various HBL-receptor k.o. mice revealed that LITAF-/- plus CDIP1-/- double k.o. mice survived 2-3 doses of HBL, while LITAF-/- plus CDIP1- /- plus CAV1-/- triple k.o. mice were fully resistant towards HBL.

This manuscript addresses a medically relevant and scientifically highly interesting topic in the field of infection diseases and toxin research. It is very clearly written and very well structured. The figures in combination with their legends are self-explanatory. The results shown are of very high quality and necessary controls, e.g. to demonstrate specificity of interactions, are included. I cannot find any weakness of this study or in this manuscript.

Our response:

We sincerely appreciate the reviewer’s highly positive and encouraging evaluation of our work. Your supportive comments regarding the clarity, structure, and rigor of the manuscript mean a great deal to us.

----------------------------------------------------------------
**Reviewer #2:**

Hemolysin BL (HBL) is a pore-forming toxin and a major virulence factor of bacillus cereus, which can cause food poisoning. The authors previously identified two proteins, LITAF and CDIP1, as key host factors (and proposed to be the receptors) for HBL toxicity through sequential genome-wide CRISPR-Cas9 screens. The authors here generated a LITAF and CDIP1 double KO mouse line and found that it showed reduced sensitivity to HBL, but not completely resistant. They therefore carried out additional genome-wide CRISPR-Cas9 screens on LITAF-CDIP1 double KO cells (using immortalized mouse embryonic fibroblast cells and also a human cell line), which identified caveolin 1 (CAV1) as another key host factor required for HBL toxicity. They showed that triple KO cells are resistant to HBL and have no binding of HBL. As CAV1 (and LITAF) is located in the inner leaflet of the membrane, the author proposed that a beta-tongue region of HBL may insert into the membrane and mediate binding to CAV1 (and LITAF). Finally, triple KO mice lacking LITAF, CDIP1, and CAV1 showed higher resistance to HBL than LITAF-CDIP1 double KO mice. These experiments are well designed and involve tremendous efforts, especially on generating knockout mouse lines, triple KO cells, and rescue cells. The role of CAV1 for HBL toxicity is novel and thoroughly validated. These findings will be interesting to a broad range of readers and will be impactful for the relevant fields.

The authors' data demonstrate that they are important host factors required for toxicity of HBL. However, as these proteins are not exposed on cell surfaces, and only located within the inner leaflet, alternative explanations other than being a "receptor" cannot be excluded based on current data presented, and could be further explored in the future. It will be better for authors to replace "receptor" with a more general term, such as "host factor", at least in the title and abstract. Additional discussion on other possibilities can be expanded in the Discussion section, including a more detailed discussion on the proposed roles of LITAF and CAV1 in reference 30, 47, and 48.

Our response:

We thank the reviewer for the positive feedback and thoughtful suggestions. We agree that alternative roles of CAV1 beyond serving as a receptor for HBL toxin cannot be formally ruled out. As suggested, we have therefore adopted the more general term “host factor” in the title and abstract, and we expanded the Discussion section to address additional possible functions. We also incorporated further discussion of the proposed roles of LITAF and CAV1 as described in references 43, 44, and 50 (References 47, 48, and 30 in the earlier version) in the Discussion section of the revised manuscript (P15).

**Minor points:**
Abstract: As CAV1 KO mice is not more resistant to HBL than WT mice, CAV1 is not a "critical determinant".

**Our response:**

Thank you for pointing this out. We have removed the word “critical” from the abstract.

Page 5: third paragraph: "MEF" can be "iMEF"? Last paragraph: the last sentence appears to be duplicated and this paragraph can be moved to Discussion.

Our response:

Thank you for pointing out these errors. The corrected last paragraph has been moved to the Discussion section.

Page 8 bottom: change 2000 ng/mL to 2 µg/mL?

Our response:

Thank you. 2000 ng/mL was changed to 2 µg/mL.

Page 9 first paragraph: bafilomycin A1 and ammonium chloride are not endocytosis inhibitors, but rather block acidification. There are other more specific inhibitors available for interfering with endocytosis.

Our response:

Thank you for this comment. We agree that bafilomycin A1 and ammonium chloride do not directly inhibit endocytosis but instead block vesicular acidification, and that using more specific endocytosis inhibitors could provide additional insight. To address this point, we employed a complementary and widely used approach based on the temperature sensitivity of endocytosis. Because endocytic uptake becomes negligible below 16 °C (References 30, 31), we exposed HT1080 cells and iMEFs to HBL toxin or the modified anthrax toxin (PA + FP59) at 15 °C, an endocytosis‑impermissive condition. As expected, anthrax toxin-mediated cytotoxicity, which requires endocytic internalization, was completely abolished at this temperature, whereas HBL‑induced cytolysis remained largely unaffected. These new data are included in Figs. S3C and S3D of the revised manuscript.

Figure 1C, D: in Fig1C, all WT mice died within 0.1 hours post-injection. In Fig1D, all WT mice were still alive at 12 hours post-injection. Why is the toxicity so different between IP and IV injection?

Our response:

Thank you for the comment. The difference in toxicity between IV and IP administration is due to how rapidly the toxin enters systemic circulation and which tissues it encounters first.

For IV injection, the toxin is delivered directly into the bloodstream, allowing it to immediately circulate and cause rapid intravascular hemolysis (similar to an acute hemolytic transfusion reaction) before it has time to distribute to other tissues. This likely models the scenario in which the toxin is released directly into the bloodstream during bacteremia. As a result, mortality occurs within minutes.

For IP injection, the toxin must first be absorbed from the peritoneal cavity. Although a portion enters the circulation via the portal system within ~30 minutes, some of the toxin is taken up locally or cleared before reaching systemic circulation. This delays the onset of systemic toxicity and explains why death occurs much later compared to the IV route.

While IV injection provides immediate systemic exposure, it is technically challenging to perform repeated IV dosing in mice because tail veins are small and easily damaged after the first injection. For this reason, IP administration is used when multiple injections are required.

Figure 2A: in the schematic model, the cells are labeled only as iMEF, but the screening was also performed using HT1080 cells as well.

Our response:

Thank you for pointing this out. We have updated Figure 2A in the revised manuscript to indicate that the screening was performed in both iMEF and HT1080 cells.

Figure 2C and other figures: as incubation of HBL with cells were carried out under room temperature for hours, the results reflect binding, internalization, and accumulation inside cells over time. The data do not reflect the initial binding to cells.

Our response:

Thank you for the comment. We agree that the measurements in Fig 2C reflect overall toxin association with cells, including binding, internalization, and intracellular accumulation. We have therefore replaced the term “binding” with “association” in Fig 2C and throughout the revised manuscript where appropriate.

Figure 2F: rescue with LITAF appears to restore full sensitivity? Does this mean CDIP1+CAV1 KO cells are not different from WT cells to HBL? Is it possible to detect and compare expression levels of LITAF, CDIP1, and CAV1 in KO cells and for these rescue experiments?

Our response:

Thank you for the comment. Because LITAF, CDIP1, and CAV1 can each independently mediate HBL toxin-induced toxicity, the full restoration of sensitivity upon LITAF re‑expression does not imply that CDIP1/CAV1 double‑KO cells respond to HBL toxin identically to WT cells. Rather, the strong rescue effect likely reflects the fact that exogenous LITAF is more efficiently and robustly expressed in transfected cells compared with CDIP1 or CAV1.

Figure 4B: there is a band around 50 kDa?

Our response:

The band is likely the IgG heavy chain from the rat anti-HBL-B antibody used for the IP, which is not present in the input lanes shown in Fig 5A. (Fig 4 in the earlier version is Fig 5 in the revised vesion)

Figure 5C: Mutation reduced overall binding, it does not suggest an interaction with CAV1.

Our response:

Thank you for the comment. We have revised the text to refer to “reduced toxin association with the cells” rather than “overall binding” in the revised manuscript (Fig 4C in the new version).

Figure 6: the experiment only showed that injection of 5 doses of toxins (2 ug each time) is tolerated, versus 3 doses tested on double KO mice. This seems to be a rather small change on experimental conditions. Is it possible to increase the dose of toxins?

Our response:

Thank you for this comment. Our in vivo experiments were designed using the LD_100_ dose of HBL toxin (2 µg, which is lethal to 100% of WT mice with a single injection) administered on consecutive days to clearly distinguish the survival curves of the LITAF/CDIP1‑dKO and LITAF/CDIP1/CAV1‑tKO mice. Under these conditions, the difference between the two groups was highly significant (*P* = 0.00106). Because 2 µg HBL represents a ≥LD_100_ dose, we did not perform extensive testing with doses higher than 2 µg. However, in our pilot studies, we observed that the tKO mice were able to tolerate three injections of substantially higher toxin doses (6 µg or 20 µg), further supporting their enhanced resistance.

Figure 6: for log-rank test of survival curves, post-hoc analysis or multiple-comparison correction is required when multiple groups are compared.

Our response:

Thank you for this comment. We have reanalyzed the survival curves as requested. Briefly, overall survival differences among the multiple groups were first evaluated using the log-rank (Mantel–Cox) test, which revealed significant group-level differences. We then performed post‑hoc pairwise log-rank tests with Holm correction to control the family-wise error rate. The conclusions remained consistent, although some *P* values changed slightly after adjustment. The adjusted *P* values are now included in Figure 6 in the revised manuscript, which were summarized below:

tKO vs dKO	0.00106

tKO vs WT	0.00081

tKO vs CAV1-KO	0.00260

dKO vs WT	0.00022

dKO vs CAV1-KO	0.00081

WT vs CAV1-KO	0.13361 (Not significant)

Gene symbols can follow the appropriate nomenclature rules: for mouse genes, only the first letter should be uppercase, followed by lowercase letters, e.g., Litaf.

Our response:

Thank you for pointing this out. We have now updated the manuscript to follow the appropriate human and mouse gene‑nomenclature conventions throughout.

In the Statistical analysis section of the Method, the authors mentioned Student's t-test or one-way ANOVA. However, I could not find any results analyzed by ANOVA. If ANOVA was not used, please remove it.

Our response:

Thank you for pointing this out. We have corrected the Statistical Analysis section to accurately reflect the tests used, and references to ANOVA have been removed.

----------------------------------------------------------------
**Reviewer #3:**

This is a well conducted study that seeks to identify the host receptors of Hemolysin BL (HBL), a tripartite α‑pore‑forming toxin of virulent Bacillus cereus. The authors identified earlier LITAF and CDIP1 as candidate receptors of HBL, but show here that HBL‑induced cytolysis, is not fully abrogated in mice lacking LITAF and CDIP1. They use a genome-wide CRISPR knockout screen in mouse embryonic fibroblasts and human HT1080 cells defective for LITAF and CDIP1, and identify caveolin-1 (CAV1) as a third host factor mediating HBL intoxication. The authors generate a mouse line lacking all three proteins and find it to be resistant to HBL challenge. They perform a series of co-purification and co-immunoprecipitation experiments suggesting that HBL-B interacts with CAV1. Because, membrane-embedded CAV1 still faces the host cytosol, the authors perform a series of experiments that suggest that the association between the toxin and CAV1 is mediated by the β‑tongue of HBL-B which inserts in the membrane to access its purported receptor. Together the findings support the notion that CAV1 acts as a receptor of HBL. The study is rigorously conducted and of broad interest.

Our response:

We sincerely appreciate your thoughtful and encouraging assessment of our work. Your positive feedback on the rigor and significance of our study is greatly valued by our team.

**Minor comments:**
-       Figure S9: this is the only experiment that attempts to demonstrate a direct interaction between CAV1 and HLB-B however it is buried in the supplementary material. Since both proteins change oligomerization status in the presence of DDM, these results should be taken with caution. Perhaps including HBL-B deleted for the β‑tongue could further solidify the conclusion that CAV1 is a receptor.

Our response:

Thank you for this helpful comment. As suggested, we have now included the HBL‑B β‑tongue deletion variant in the assay. Consistent with our model, WT HBL‑B associates with CAV1, whereas the β‑tongue deletion variant fails to do so. The results are presented in the revised manuscript as S11 Fig.

-       Alternatively in Figure S8, the authors could show immunoprecipitation results in the presence of DTSSP and omit the reducing agent in the western blot analysis. Arguably the minimal CAV1 and HLB-B complex might be larger than the sum of the two monomers.

Our response:

Thank you for this constructive suggestion. Because releasing immunoprecipitated material from antibody-conjugated magnetic beads requires non‑native conditions (e.g., heating in SDS sample loading buffer), it is technically challenging to perform this experiment under fully native, non‑reducing conditions. For this reason, we pursued the alternative approach described above to address this issue. (Fig S8 is S10 Fig in the revised version)

-       Figure S8: The data shows that cross-linking is not required to stabilize co-immunoprecipitants of CAV1 and HLB-B but the main text remains silent on the matter.

Our response:

Thank you for this constructive comment. We have now added a brief statement in the Results and Discussion sections (P12, P14 and P15) to reflect this fact in the revised manuscript.

-       I wonder if the authors can elaborate on the fact that LITAF and CDIP1 can serve as receptors for HLB.  CDIP1 has a preferential endosome/lysosome membrane localization and like CAV1, LITAF association with the cytoplasmic membrane, puts the protein fully facing inside the cell.  Is the β‑tongue of HBL-B also required for LITAF/CDIP1 interactions? Data in Fig 5C seems to indicate that it is the case.

Our response:

Thank you for this constructive comment. We have expanded the discussion to further clarify how LITAF and CDIP1 can function as host factors for HBL. As both proteins are localized to the inner leaflet of the plasma membrane, their orientation is similar to that of CAV1. Consistent with this, the data in now Fig. 4C indicate that the β‑tongue region of HBL‑B is also required for interactions with LITAF/CDIP1. We have elaborated on this point in the revised manuscript (P11 and P14).

Additional comments for the authors consideration:
-       Intoxication with HBL in animals: I wonder if the authors can comment on why they opt for IP or IV delivery of the toxin. Presumably Bacillus cereus is ingested. Is the assumption here that HBL becomes important for disease only after GI barriers have been breached? Or it is assumed that HBL crosses the GI epithelium? The authors cite references 16 and 17 to substantiate that HLB is a well-established virulence factor but only reference 17 uses bacteria for infection and in this reference bacteria (WT versus HLB mutants are compared) are delivered IP bypassing GI tract breach.

Our response:

Thank you for these insightful comments. We have expanded the Methods section (Mouse studies) to clarify the rationale for using IP or IV delivery of HBL in our experimental models and to better contextualize the role of HBL during *Bacillus cereus* infection. Although *B. cereus* is typically acquired through ingestion, purified HBL is unlikely to remain stable or active within the protease‑rich gastrointestinal environment. For this reason, oral administration of the toxin would not be expected to recapitulate its pathogenic potential. Instead, our IP/IV models are intended to mimic stages of infection in which *B. cereus* has already breached epithelial barriers or disseminated systemically, conditions under which HBL becomes biologically relevant.

We agree that HBL is likely to play a more prominent role once gastrointestinal barriers have been compromised. *B. cereus* is an opportunistic pathogen capable of causing severe intestinal disease, particularly in immunocompromised individuals. In documented cases of bacteremia and systemic infection (References 4–9), HBL has been implicated as a major contributor to host tissue damage and lethality. Thus, once the bacteria reach the bloodstream or deep tissues, HBL is positioned to exert its cytolytic activity and functions as a critical virulence factor.

Beyond systemic disease, *B. cereus* is well known for causing fulminant ocular infections, in which HBL plays a central role in rapid tissue destruction and irreversible vision loss (Reference 9). These clinical contexts underscore that HBL’s pathogenic importance extends beyond the gastrointestinal tract and is most pronounced when physical barriers are bypassed or compromised.

We have expanded the Discussion section to incorporate these points (P16).

-       Can the authors fully rule out that CAV1, LITAF, CDIP1 are the receptors and not part of a trafficking machinery important for toxin delivery inside the cell?

Our response:

Thank you for this comment. This is a shared comment also raised by Reviewer #2. Although we could not formally rule out this possibility, it is highly unlikely that they are part of a trafficking machinery important for toxin delivery inside the cell, due to the following observations: (1) Like other pore‑forming toxins, HBL induces plasma membrane blebbing within minutes, indicating that the plasma membrane is its primary target. (2) Receptor‑mediated toxin internalization and trafficking are temperature‑sensitive processes that are largely inhibited between 4 °C and 16 °C; under these conditions, anthrax toxin fails to intoxicate cells. In contrast, HBL remains fully active in plasma membrane pore formation at low temperatures, demonstrating that its cytolytic activity is temperature‑insensitive and therefore unlikely to depend on endocytic trafficking. These new data were included in S3C and S3D Figs in the revised manuscript and discussed in the Discussion section (P16).

-       Can the authors comment on the selectivity of the toxin? It seems that all the cells can be targeted by HLB. How does that correlate with disease following bacterial infection?

Our response:

Thank you for raising this important point. All primary cells and cell lines examined to date are susceptible to HBL toxin (Reference 18), indicating that they express at least one member of this potential receptor set (LITAF, CDIP1, and CAV1). This lack of strict cellular specificity, consistent with the broad expression of LITAF, CDIP1, and CAV1 (The Human Protein Atlas), is not unusual among pore‑forming toxins, which constitute one of the largest and most potent classes of bacterial virulence factors. Although it remains unclear why *B. cereus* evolved a toxin with such broad activity, its non‑selective cytolytic capacity aligns with the clinical manifestations observed once the bacteria breach local barriers. During systemic infection or bacteremia, HBL’s ability to damage diverse host cell types, including epithelial, endothelial, and immune cells, would be expected to accelerate tissue injury, promote vascular leakage, and compromise host defense mechanisms. These combined effects likely contribute to the rapid progression and severity of B. cereus infections in extra‑intestinal settings (References 4-9).

We have clarified this point in the revised Discussion to better connect HBL’s broad cellular tropism with its pathogenic role during disseminated infection (P16).
