# Supplementary material for: Caveolin 1 is a conserved host factor mediating Bacillus cereus hemolysin BL toxin cytolytic toxicity
Source: PLoS Biol. 2026 Aug 21;24(8):e3003957. doi: 10.1371/journal.pbio.3003957 (PMC13524335; doi:10.1371/journal.pbio.3003957)
Supplement: Peer review history

# Editor report: Decision Letter 0

**DOI:** 10.1371/journal.pbio.3003957.r001

**Authors:** Melissa Vazquez Hernandez

17 Apr 2026

Dear Dr Liu,

Thank you for submitting your manuscript entitled "Caveolin 1 is a conserved host receptor mediating Bacillus cereus hemolysin BL toxin cytolytic toxicity" for consideration as a Research Article by PLOS Biology.

Your manuscript has now been evaluated by the PLOS Biology editorial staff as well as by an academic editor with relevant expertise and I am writing to let you know that we would like to send your submission out for external peer review.

However, before we can send your manuscript to reviewers, we need you to complete your submission by providing the metadata that is required for full assessment. To this end, please login to Editorial Manager where you will find the paper in the 'Submissions Needing Revisions' folder on your homepage. Please click 'Revise Submission' from the Action Links and complete all additional questions in the submission questionnaire.

Once your full submission is complete, your paper will undergo a series of checks in preparation for peer review. After your manuscript has passed the checks it will be sent out for review. To provide the metadata for your submission, please Login to Editorial Manager (https://www.editorialmanager.com/pbiology) within two working days, i.e. by Apr 19 2026 11:59PM.

If your manuscript has been previously peer-reviewed at another journal, PLOS Biology is willing to work with those reviews in order to avoid re-starting the process. Submission of the previous reviews is entirely optional and our ability to use them effectively will depend on the willingness of the previous journal to confirm the content of the reports and share the reviewer identities. Please note that we reserve the right to invite additional reviewers if we consider that additional/independent reviewers are needed, although we aim to avoid this as far as possible. In our experience, working with previous reviews does save time.

If you would like us to consider previous reviewer reports, please edit your cover letter to let us know and include the name of the journal where the work was previously considered and the manuscript ID it was given. In addition, please upload a response to the reviews as a 'Prior Peer Review' file type, which should include the reports in full and a point-by-point reply detailing how you have or plan to address the reviewers' concerns.

During the process of completing your manuscript submission, you will be invited to opt-in to posting your pre-review manuscript as a bioRxiv preprint. Visit http://journals.plos.org/plosbiology/s/preprints for full details. If you consent to posting your current manuscript as a preprint, please upload a single Preprint PDF.

Feel free to email us at plosbiology@plos.org if you have any queries relating to your submission.

Kind regards,

Melissa

Melissa Vazquez Hernandez, Ph.D.

Associate Editor

PLOS Biology

mvazquezhernandez@plos.org

# Peer review: Decision Letter 1

**DOI:** 10.1371/journal.pbio.3003957.r002

**Authors:** Melissa Vazquez Hernandez

15 Jun 2026

Dear Dr Liu,

Thank you for your patience while your manuscript "Caveolin 1 is a conserved host receptor mediating Bacillus cereus hemolysin BL toxin cytolytic toxicity" went through peer-review at PLOS Biology. Your manuscript has now been evaluated by the PLOS Biology editors, an Academic Editor with relevant expertise, and by three independent reviewers.

As you will see from the reports, all three reviewers are supportive and agree that the study is carefully executed and of broad interest. We are therefore inviting a light major revision to address a small number of points that are important for further consideration. Most importantly, reviewers #2 and #3 ask that you clarify or moderate the “receptor” claim, given the cytosolic/inner-leaflet localization of CAV1, LITAF, and CDIP1, and expand the Discussion to consider alternative host-factor roles. Please also consider reviewer #3’s suggestions for strengthening the direct-interaction evidence, including the HBL-B β-tongue deletion mutant in the Fig. S9 assay and/or non-reducing crosslinking analysis in Fig. S8, or explain why these experiments are not feasible. Please also address all the remaining minor concerns from the reviewers.

In addition to these revisions, you may need to complete some formatting changes, which you will receive in a follow up email. A member of our team will be in touch with a set of requests shortly. If you do not receive a separate email within a few days, please assume that checks have been completed, and no additional changes are required.

We expect to receive your revised manuscript within 1 month. Please email us (plosbiology@plos.org) if you have any questions or concerns, or would like to request an extension.

At this stage, your manuscript remains formally under active consideration at our journal; please notify us by email if you do not intend to submit a revision so that we withdraw the manuscript.

**IMPORTANT - SUBMITTING YOUR REVISION**

Your revisions should address the specific points made by each reviewer. Please submit the following files along with your revised manuscript:

1. A 'Response to Reviewers' file - this should detail your responses to the editorial requests, present a point-by-point response to all of the reviewers' comments, and indicate the changes made to the manuscript.

*NOTE: In your point-by-point response to the reviewers, please provide the full context of each review. Do not selectively quote paragraphs or sentences to reply to. The entire set of reviewer comments should be present in full and each specific point should be responded to individually.

You should also cite any additional relevant literature that has been published since the original submission and mention any additional citations in your response.

2. In addition to a clean copy of the manuscript, please also upload a 'track-changes' version of your manuscript that specifies the edits made. This should be uploaded as a "Revised Article with Changes Highlighted " file type.

*Resubmission Checklist*

When you are ready to resubmit your revised manuscript, please refer to this resubmission checklist: https://plos.io/Biology_Checklist

To submit a revised version of your manuscript, please go to https://www.editorialmanager.com/pbiology/ and log in as an Author. Click the link labelled 'Submissions Needing Revision' where you will find your submission record.

Please make sure to read the following important policies and guidelines while preparing your revision:

*Published Peer Review*

Please note while forming your response, if your article is accepted, you may have the opportunity to make the peer review history publicly available. The record will include editor decision letters (with reviews) and your responses to reviewer comments. If eligible, we will contact you to opt in or out. Please see here for more details:

https://blogs.plos.org/plos/2019/05/plos-journals-now-open-for-published-peer-review/

*PLOS Data Policy*

Please note that as a condition of publication PLOS' data policy (http://journals.plos.org/plosbiology/s/data-availability) requires that you make available all data used to draw the conclusions arrived at in your manuscript. If you have not already done so, you must include any data used in your manuscript either in appropriate repositories, within the body of the manuscript, or as supporting information (N.B. this includes any numerical values that were used to generate graphs, histograms etc.). For an example see here: http://www.plosbiology.org/article/info%3Adoi%2F10.1371%2Fjournal.pbio.1001908#s5

*Blot and Gel Data Policy*

We require the original, uncropped and minimally adjusted images supporting all blot and gel results reported in an article's figures or Supporting Information files. We will require these files before a manuscript can be accepted so please prepare them now, if you have not already uploaded them. Please carefully read our guidelines for how to prepare and upload this data: https://journals.plos.org/plosbiology/s/figures#loc-blot-and-gel-reporting-requirements

*Protocols deposition*

To enhance the reproducibility of your results, we recommend that if applicable you deposit your laboratory protocols in protocols.io, where a protocol can be assigned its own identifier (DOI) such that it can be cited independently in the future. Additionally, PLOS ONE offers an option for publishing peer-reviewed Lab Protocol articles, which describe protocols hosted on protocols.io. Read more information on sharing protocols at https://plos.org/protocols?utm_medium=editorial-email&utm_source=authorletters&utm_campaign=protocols

Thank you again for your submission to our journal. We hope that our editorial process has been constructive thus far, and we welcome your feedback at any time. Please don't hesitate to contact us if you have any questions or comments.

Sincerely,

Melissa

Melissa Vazquez Hernandez, Ph.D.

Associate Editor

PLOS Biology

mvazquezhernandez@plos.org

----------------------------------------------------------------

REVIEWERS' COMMENTS

----------------------------------------------------------------

Reviewer #1 (Holger Barth):

In the present manuscript, the authors report about the identification and characterization of a third cellular receptor for the tripartite α‑pore‑forming toxin and a major virulence factor hemolysin BL (HBL) from the medically relevant human pathogenic bacterium Bacillus cereus. Before, this consortium has identified and characterized two other cellular membrane receptors for HBL, namely LITAF and CDIP1. They recognized that cells lacking these two receptors were still intoxicated by exposure to HBL, although not as efficient as wild-type cells. This prompted the authors to use double-knock out mouse and human cells to perform genome-wide CRISPR knockout screens to identify cells that are totally resistant towards HBL. The only hit found in these cells was caveolin 1 (CAV1), a trans-membrane protein. The authors then performed a elegant series of experiments including co-localization by fluorescence microsocpy and co-preciptation / Western blot analyis to investigate whether the binding subunit of HBL (HBL-B) interacts with CAV1 in cells, which was convincingly demonstarted by the results. All necessary controls were involved in these experiments and the conclusions drawn from these results are justified. Next, the interaction between HBL-B and Cav1 was analyzed in more deatil. To this end, specific amino acid residues in the so-called ß-tongue region of HBL-B were modified and the resulting variations charactierzed and tested for binding to CAv-1 and for cytotoxicity of cells. Taken together, the importance of the ß-tongue for binding to CAV-1, which is mainly localized on the inner side of the plasma membrane was confirmed and residues I245, G246 and I 248 identified as crucial for the binding of HBL-B to Cav-1. Again, all necessary controls were included and the results are convincing enough to support the conclusions. Amongst other detailed characterizations of the interction of HBL with Cav1 and the other receptors, the authors also excluded that CAV-1 interacts with the other two HBL receptors in cells. Finally and importantly, the role of CAV-1 for HBL pathogenesis was confirmed in vivo by using a mouse model. Interestingly, the results obtained with various HBL-receptor k.o. mice revealed that LITAF-/- plus CDIP1-/- double k.o. mice survived 2-3 doses of HBL, while LITAF-/- plus CDIP1- /- plus CAV1-/- triple k.o. mice were fully resistant towards HBL.

This manuscript addresses a medically relevant and scientifically highy interesting topic in the field of infection diseases and toxin research. It is very clearly written and very well structured. The figures in combination with their legends are self-explanatory. The results shown are of very high quality and necessary controls, e.g. to demonstratee specificity of interctions, are included. I can not find any weaknes of this study or in this manuscript.

----------------------------------------------------------------

Reviewer #2:

Hemolysin BL (HBL) is a pore-forming toxin and a major virulence factor of bacillus cereus, which can cause food poisoning. The authors previously identified two proteins, LITAF and CDIP1, as key host factors (and proposed to be the receptors) for HBL toxicity through sequential genome-wide CRISPR-Cas9 screens. The authors here generated a LITAF and CDIP1 double KO mouse line and found that it showed reduced sensitivity to HBL, but not completely resistant. They therefore carried out additional genome-wide CRISPR-Cas9 screens on LITAF-CDIP1 double KO cells (using immortalized mouse embryonic fibroblast cells and also a human cell line), which identified caveolin 1 (CAV1) as another key host factor required for HBL toxicity. They showed that triple KO cells are resistant to HBL and have no binding of HBL. As CAV1 (and LITAF) is located in the inner leaflet of the membrane, the author proposed that a beta-tongue region of HBL may insert into the membrane and mediate binding to CAV1 (and LITAF). Finally, triple KO mice lacking LITAF, CDIP1, and CAV1 showed higher resistance to HBL than LITAF-CDIP1 double KO mice. These experiments are well designed and involve tremendous efforts, especially on generating knockout mouse lines, triple KO cells, and rescue cells. The role of CAV1 for HBL toxicity is novel and thoroughly validated. These findings will be interesting to a broad range of readers and will be impactful for the relevant fields.

The authors' data demonstrate that they are important host factors required for toxicity of HBL. However, as these proteins are not exposed on cell surfaces, and only located within the inner leaflet, alternative explanations other than being a "receptor" cannot be excluded based on current data presented, and could be further explored in the future. It will be better for authors to replace "receptor" with a more general term, such as "host factor", at least in the title and abstract. Additional discussion on other possibilities can be expanded in the Discussion section, including a more detailed discussion on the proposed roles of LITAF and CAV1 in reference 30, 47, and 48.

Minor points:

Abstract: As CAV1 KO mice is not more resistant to HBL than WT mice, CAV1 is not a "critical determinant".

Page 5: third paragraph: "MEF" can be "iMEF"? Last paragraph: the last sentence appears to be duplicated and this paragraph can be moved to Discussion.

Page 8 bottom: change 2000 ng/mL to 2 ug/mL?

Page 9 first paragraph: bafilomycin A1 and ammonium chloride are not endocytosis inhibitors, but rather block acidification. There are other more specific inhibitors available for interfering with endocytosis.

Figure 1C,D: in Fig1C, all WT mice died within 0.1 hours post-injection. In Fig1D, all WT mice were still alive at 12 hours post-injection. Why is the toxicity so different between IP and IV injection?

Figure 2A: in the schematic model, the cells are labeled only as iMEF, but the screening was also performed using HT1080 cells as well.

Figure 2C and other figures: as incubation of HBL with cells were carried out under room temperature for hours, the results reflect binding, internalization, and accumulation inside cells over time. The data do not reflect the initial binding to cells.

Figure 2F: rescue with LITAF appears to restore full sensitivity? Does this mean CDIP1+CAV1 KO cells are not different from WT cells to HBL? Is it possible to detect and compare expression levels of LITAF, CDIP1, and CAV1 in KO cells and for these rescue experiments?

Figure 4B: there is a band around 50 kDa?

Figure 5C: Mutation reduced overall binding, it does not suggest an interaction with CAV1.

Figure 6: the experiment only showed that injection of 5 doses of toxins (2 ug each time) is tolerated, versus 3 doses tested on double KO mice. This seems to be a rather small change on experimental conditions. Is it possible to increase the dose of toxins?

Figure 6: for log-rank test of survival curves, post-hoc analysis or multiple-comparison correction is required when multiple groups are compared.

Gene symbols can follow the appropriate nomenclature rules: for mouse genes, only the first letter should be uppercase, followed by lowercase letters, e.g., Litaf.

In the Statistical analysis section of the Method, the authors mentioned Student's t-test or one-way ANOVA. However, I could not find any results analyzed by ANOVA. If ANOVA was not used, please remove it.

----------------------------------------------------------------

Reviewer #3:

This is a well conducted study that seeks to identify the host receptors of Hemolysin BL (HBL), a tripartite α‑pore‑forming toxin of virulent Bacillus cereus. The authors identified earlier LITAF and CDIP1 as candidate receptors of HBL, but show here that HBL‑induced cytolysis, is not fully abrogated in mice lacking LITAF and CDIP1. They use a genome-wide CRISPR knockout screen in mouse embryonic fibroblasts and human HT1080 cells defective for LITAF and CDIP1, and identify caveolin-1 (CAV1) as a third host factor mediating HBL intoxication. The authors generate a mouse line lacking all three proteins and find it to be resistant to HBL challenge. They perform a series of co-purification and co-immunoprecipitation experiments suggesting that HBL-B interacts with CAV1. Because, membrane-embedded CAV1 still faces the host cytosol, the authors perform a series of experiments that suggest that the association between the toxin and CAV1 is mediated by the β‑tongue of HBL-B which inserts in the membrane to access its purported receptor. Together the findings support the notion that CAV1 act as a receptor of HBL. The study is rigorously conducted and of broad interest.

Minor comments:

-Figure S9: this is the only experiment that attempts to demonstrate a direct interaction between CAV1 and HLB-B however it is buried in the supplementary material. Since both proteins change oligomerization status in the presence of DDM, these results should be taken with caution. Perhaps including HBL-B deleted for the β‑tongue could further solidify the conclusion that CAV1 is a receptor.

-Alternatively in Figure S8, the authors could show immunoprecipitation results in the presence of DTSSP and omit the reducing agent in the western blot analysis. Arguably the minimal CAV1 and HLB-B complex might be larger than the sum of the two monomers.

-Figure S8: The data shows that cross-linking is not required to stabilize co-immunoprecipitants of CAV1 and HLB-B but the main text remains silent on the matter.

-I wonder if the authors can elaborate on the fact that LITAF and CDIP1 can serve as receptors for HLB. CDIP1 has a preferential endosome/lysosome membrane localization and like CAV1, LITAF association with the cytoplasmic membrane, puts the protein fully facing inside the cell. Is the β‑tongue of HBL-B also required for LITAF/CDIP1 interactions? Data in Fig 5C seem to indicate that it is the case.

Additional comments for the authors consideration:

-Intoxication with HBL in animals: I wonder if the authors can comment why they opt for IP or IV delivery of the toxin. Presumably Bacillus cereus is ingested. Is the assumption here that HBL becomes important for disease only after GI barriers have been breached? Or it is assumed that HBL crosses the GI epithelium? The authors cite references 16 and 17 to substantiate that HLB is a well-established virulence factor but only reference 17 uses bacteria for infection and in this reference bacteria (WT versus HLB mutants are compared) are delivered IP bypassing GI tract breach.

-Can the authors fully rule out that CAV1, LITAF, CDIP1 are the receptors and not part of a trafficking machinery important for toxin delivery inside the cell?

-Can the authors comment on the selectivity of the toxin? It seems that all the cells can be targeted by HLB. How does that correlate with disease following bacterial infection?

# Author comment: Author response to Decision Letter 2

**DOI:** 10.1371/journal.pbio.3003957.r003

16 Jul 2026

Attachment Submitted filename: Point-by-point responses to the reviewers 7.14.26.docx

# Editor report: Decision Letter 2

**DOI:** 10.1371/journal.pbio.3003957.r004

**Authors:** Melissa Vazquez Hernandez

29 Jul 2026

Dear Shihui,

Thank you for your patience while we considered your revised manuscript "Caveolin 1 is a conserved host factor mediating Bacillus cereus hemolysin BL toxin cytolytic toxicity" for publication as a Research Article at PLOS Biology. This revised version of your manuscript has been evaluated by the PLOS Biology editors, and the Academic Editor.

Based on our Academic Editor's assessment of your revision, we are likely to accept this manuscript for publication, provided you satisfactorily address the following data and other policy-related requests.

1) Please add the weblink of the funding agencies in the Financial Disclosure statement in the manuscript details.

2) Thank you for providing the raw data underlying the figures. Please cite the location of the data clearly in all relevant main and supplementary Figure legends, e.g. “The data underlying this Figure can be found in S1 Data” or “The data underlying this Figure can be found in https://doi.org/10.5281/zenodo.XXXXX”

3) Supplementary files (e.g., excel). Please ensure that all data files are uploaded as 'Supporting Information' and are invariably referred to (in the manuscript, figure legends, and the Description field when uploading your files) using the following format verbatim: S1 Data, S2 Data, etc. Multiple panels of a single or even several figures can be included as multiple sheets in one excel file that is saved using exactly the following convention: S1_Data.xlsx (using an underscore).

4) Please add a scale bar in the following microscopy pictures in Figures: S6AB

5) Please ensure that your Data Statement in the submission system accurately describes where your data can be found and is in final format, as it will be published as written there

6) Per journal policy, if you have generated any custom code during the course of this investigation, please make it available without restrictions. Please ensure that the code is sufficiently well documented and reusable, and that your Data Statement in the Editorial Manager submission system accurately describes where your code can be found. More information on our Code Policy, what and how to share can be found here: https://journals.plos.org/plosbiology/s/code-availability

Please note that we cannot accept sole deposition of code in GitHub, as this could be changed after publication. However, you can archive this version of your publicly available GitHub code to Zenodo. Once you do this, it will generate a DOI number, which you will need to provide in the Data Accessibility Statement (you are welcome to also provide the GitHub access information). See the process for doing this here: https://docs.github.com/en/repositories/archiving-a-github-repository/referencing-and-citing-content

As you address these items, please take this last chance to review your reference list to ensure that it is complete and correct. If you have cited papers that have been retracted, please include the rationale for doing so in the manuscript text, or remove these references and replace them with relevant current references. Any changes to the reference list should be mentioned in the cover letter that accompanies your revised manuscript.

In addition to these revisions, you may need to complete some formatting changes, which you will receive in a follow up email. A member of our team will be in touch with a set of requests shortly. If you do not receive a separate email within a few days, please assume that checks have been completed, and no additional changes are required.

We expect to receive your revised manuscript within two weeks.

To submit your revision, please go to https://www.editorialmanager.com/pbiology/ and log in as an Author. Click the link labelled 'Submissions Needing Revision' to find your submission record. Your revised submission must include the following:

- a cover letter that should detail your responses to any editorial requests, if applicable, and whether changes have been made to the reference list

- a Response to Reviewers file that provides a detailed response to the reviewers' comments (if applicable, if not applicable please do not delete your existing 'Response to Reviewers' file.)

- a track-changes file indicating any changes that you have made to the manuscript.

NOTE: If Supporting Information files are included with your article, note that these are not copyedited and will be published as they are submitted. Please ensure that these files are legible and of high quality (at least 300 dpi) in an easily accessible file format. For this reason, please be aware that any references listed in an SI file will not be indexed. For more information, see our Supporting Information guidelines:

https://journals.plos.org/plosbiology/s/supporting-information

*Published Peer Review History*

Please note that you may have the opportunity to make the peer review history publicly available. The record will include editor decision letters (with reviews) and your responses to reviewer comments. If eligible, we will contact you to opt in or out. Please see here for more details:

https://plos.org/published-peer-review-history/

*Press*

Should you, your institution's press office or the journal office choose to press release your paper, please ensure you have opted out of Early Article Posting on the submission form. We ask that you notify us as soon as possible if you or your institution is planning to press release the article.

*Protocols deposition*

To enhance the reproducibility of your results, we recommend that if applicable you deposit your laboratory protocols in protocols.io, where a protocol can be assigned its own identifier (DOI) such that it can be cited independently in the future. Additionally, PLOS ONE offers an option for publishing peer-reviewed Lab Protocol articles, which describe protocols hosted on protocols.io. Read more information on sharing protocols at https://plos.org/protocols?utm_medium=editorial-email&utm_source=authorletters&utm_campaign=protocols

Please do not hesitate to contact me should you have any questions.

Sincerely,

Melissa

Melissa Vazquez Hernandez, Ph.D.

Senior Editor

mvazquezhernandez@plos.org

PLOS Biology

# Author comment: Author response to Decision Letter 3

**DOI:** 10.1371/journal.pbio.3003957.r005

3 Aug 2026

Attachment Submitted filename: Point-by-point_responses_to_the_reviewers_7.14.26_auresp_3.docx

# Editor report: Decision Letter 3

**DOI:** 10.1371/journal.pbio.3003957.r006

**Authors:** Melissa Vazquez Hernandez

5 Aug 2026

Dear Shihui,

I hope you are doing great and enjoying the summer!

Thank you for the submission of your revised Research Article "Caveolin 1 is a conserved host factor mediating Bacillus cereus hemolysin BL toxin cytolytic toxicity" for publication in PLOS Biology. On behalf of my colleagues and the Academic Editor, Sebastian Winter, I am pleased to say that we can in principle accept your manuscript for publication, provided you address any remaining formatting and reporting issues. These will be detailed in an email you should receive within 2-3 business days from our colleagues in the journal operations team; no action is required from you until then. Please note that we will not be able to formally accept your manuscript and schedule it for publication until you have completed any requested changes.

Please take a minute to log into Editorial Manager at http://www.editorialmanager.com/pbiology/, click the "Update My Information" link at the top of the page, and update your user information to ensure an efficient production process.

PRESS

We frequently collaborate with press offices. If your institution or institutions have a press office, please notify them about your upcoming paper at this point, to enable them to help maximise its impact. If the press office is planning to promote your findings, we would be grateful if they could coordinate with biologypress@plos.org. If you have previously opted in to the early version process, we ask that you notify us immediately of any press plans so that we may opt out on your behalf.

We also ask that you take this opportunity to read our Embargo Policy regarding the discussion, promotion and media coverage of work that is yet to be published by PLOS. As your manuscript is not yet published, it is bound by the conditions of our Embargo Policy. Please be aware that this policy is in place both to ensure that any press coverage of your article is fully substantiated and to provide a direct link between such coverage and the published work. For full details of our Embargo Policy, please visit http://www.plos.org/about/media-inquiries/embargo-policy/.

Thank you again for choosing PLOS Biology for publication and supporting Open Access publishing. We look forward to publishing your study.

Sincerely,

Melissa

Melissa Vazquez Hernandez, Ph.D., Ph.D.

Senior Editor

PLOS Biology

mvazquezhernandez@plos.org
